# Lack of ethics or lack of knowledge? European upper secondary students’ doubts and misconceptions about integrity issues

**DOI:** 10.1007/s40979-022-00113-0

**Published:** 2022-08-11

**Authors:** Mikkel Willum Johansen, Mads Paludan Goddiksen, Mateja Centa, Christine Clavien, Eugenijus Gefenas, Roman Globokar, Linda Hogan, Marcus Tang Merit, Søren Saxmose Nielsen, I. Anna S. Olsson, Margarita Poškutė, Una Quinn, Júlio Borlido Santos, Rita Santos, Céline Schöpfer, Vojko Strahovnik, P. J. Wall, Peter Sandøe, Thomas Bøker Lund

**Affiliations:** 1 Department of Science Education, University of Copenhagen, Copenhagen, Denmark; 2 Department of Food and Resource Economics, University of Copenhagen, Copenhagen, Denmark; 3 Faculty of Theology, University of Ljubljana, Ljubljana, Slovenia; 4 Institute for Ethics, History and the Humanities, Faculty of Medicine, University of Geneva, Geneva, Switzerland; 5 Centre for Health Ethics, Law and History, Institute of Health Sciences, Faculty of Medicine, Vilnius University, Vilnius, Lithuania; 6 School of Ecumenics, Trinity College Dublin, Dublin, Ireland; 7 Institute of Architecture, Urbanism and Landscape, Royal Danish Academy, Copenhagen, Denmark; 8 Department of Veterinary and Animal Sciences, University of Copenhagen, Copenhagen, Denmark; 9 i3S – Instituto de Investigação e Inovação em Saúde, Universidade do Porto, Rua Alfredo Allen 208, 4200-135 Porto, Portugal; 10 Faculty of Arts, University of Ljubljana, Ljubljana, Slovenia; 11 ADAPT Centre, School of Computer Science and Statistics, Trinity College Dublin, Dublin, Ireland

**Keywords:** Academic integrity, Questionable academic practices, Upper secondary students, Plagiarism, Training

## Abstract

Plagiarism and other transgressions of the norms of academic integrity appear to be a persistent problem among upper secondary students. Numerous surveys have revealed high levels of infringement of what appear to be clearly stated rules. Less attention has been given to students’ understanding of academic integrity, and to the potential misconceptions and false beliefs that may make it difficult for them to comply with existing rules and handle complex real-life situations.

In this paper we report findings from a survey of European upper secondary students’ views on issues relating to academic integrity. We relate these findings to the students’ training about academic integrity, self-reported level of questionable behavior and country of study.

A total of 1654 students at 51 institutions located in 6 European countries participated in the study. The participants generally believed they had a good understanding of the rules applying to them and knew how to behave in compliance with norms of academic integrity. The results indicate, however, that often, in practice, this belief was mistaken. Many students had an inadequate understanding of core elements of academic integrity. They were uncertain about how to act, and they struggled in the handling of complex situations that require context-sensitive judgement. While some differences between countries were identified, they were modest and exhibited no clear pattern. Our results also suggest that reducing students’ level of uncertainty and, to a lesser degree, improving their level of knowledge could lead them to engage less in certain types of questionable behaviours. Surprisingly, the effect of academic training is modest and ambiguous. The study also confirms that perception of peer behaviour has the strongest association with student engagement in questionable behaviours. Thus, academic integrity at the upper secondary level cannot be explained simply in terms of individual ethics or knowledge.

**Supplementary Information:**

The online version contains supplementary material available at 10.1007/s40979-022-00113-0.

## Introduction

Plagiarism and other transgressions of the norms of academic integrity appear to be widespread among upper secondary students. In an early investigation, Schab (1991) found high and generally increasing levels in self-reported levels of behaviours such as plagiarism (from 67% in 1969 to 76% in 1989), the use of cheat sheets (from 34% in 1969 to 68% in 1989) and handing in work done by another student (from 50.5% in 1969 to 51.5% in 1989). In their 2000 survey of 2342 American upper secondary students, McCabe et al. found that, depending on the type of school, 67–81% of the students admitted that they had plagiarised or cheated on a test (McCabe et al. 2012, p. 22). Similar results have been reported in other surveys of American upper secondary students (e.g., Josephson Institute of Ethics 2012, pp. 46, Galloway 2012; see Davis et al. 2009, pp. 35 for an overview). Comparable levels of dishonesty among upper secondary students have been reported from several other countries, including Spain (Sureda-Negre et al. 2015), Indonesia (Pramadi et al. 2017) and Slovenia (Šorgo et al. 2015). Widespread cheating and corruption has been reported among Cambodian secondary students (Brehm 2016), while a survey of Taiwanese students reported markedly lower levels of plagiarism (Chang et al. 2015).

There are several good reasons to promote academic integrity already at the upper secondary level. Breaches of rules and norms relating to academic integrity make it difficult for institutions to evaluate the students fairly, and widespread breaches may create a race to the bottom, where students feel compelled to cheat or plagiarise to compete with their peers (McCabe 1999; McCabe et al. 2012: 27). In other words, infringements of the rules of academic integrity can create an insecure and cynical learning environment. Furthermore, the transgressive behaviours a student learns at an earlier stage of the educational trajectory may be transferred to later stages or even to the student’s professional life (Baldwin Jr et al. 1996; Harding et al. 2004; Ip et al. 2016; Guerrero-Dib et al. 2020). Thus, learning sound academic behaviours already, at upper secondary level, may improve the students’ behaviour not only at that level but throughout their education and beyond.

It is, however, not clear how best to improve academic integrity behaviour among students. In the existing literature lack of integrity is often framed as an ethical or motivational (rather than an epistemic) problem (Fishman 2016). This underlying perspective on the problem is seen in the frequent use of words such as ‘cheating’ and ‘dishonesty’ to describe breaches of the rules, or norms, of academic integrity. In line with this conceptualisation of the problem, some of the solutions suggested in the literature involve either more effective policing (e.g., using plagiarism detection software (Villano 2006)), or the improvement of students’ ethical awareness (e.g., by the establishment of honour codes, (McCabe et al. 2012), or ethical character building (Stephens and Wangaard 2013)).

While wilful transgression of rules and norms may represent an important part of the current problem of academic integrity, there are undoubtedly other important factors behind the patterns of behaviour we see. Students at all levels are known to justify their transgressions by using various neutralization strategies, such as claiming that an assignment was pointless or that they were given too much homework (e.g., McCabe 1992; Stephens and Gehlbach 2007; Zito and McQuillan 2011; Stephens 2018). Thus although a student may know that an action is wrong she may do it anyway because her sense of responsibility has been undermined through neutralization. Relatedly, students’ ethical behaviour has been found to be directly correlated with their perception of the behaviour of their peers (McCabe and Treviño 1993; McCabe et al. 2001; O’Fallon and Butterfield 2012). Although this effect has been studied mainly in tertiary education there is evidence that it is also present at the upper secondary level (Stephens and Gehlbach 2007). In other words, students who contravene rules and norms may may be rationalizing their actions to themselves with the belief that everybody else is transgressing the norms as well or by denying their own responsibility.

In these situations, students are assumed to have some knowledge of the rules and norms they transgress. However, very little attention has been given to situations where students break the rules or norms because they have misunderstood what is required of them. Similarly, limited thought has been given to tricky grey-zone situations that call for context-sensitive judgements rather than blind application of clear, general rules.

Investigations of undergraduate university students (i.e. those one level above the upper secondary level with which we are here concerned) have shown a clear discrepancy between the way students and their teachers rate the seriousness of various transgressions of the rules (Franklyn-Stokes and Newstead 1995; McCabe et al. 2012, p. 31). Furthermore, undergraduate students’ understanding of central concepts of academic integrity has been found to be underdeveloped. Thus, in a test performed by Miguel Roig, students had great difficulty distinguishing between acceptable and unacceptable paraphrases of a given text (Roig 1997). In a natural experiment in which undergraduate students were given a tutorial on plagiarism it was found that the tutorial significantly reduced their likelihood of plagiarizing, apparently by improving their understanding of plagiarism rather than by raising their fear of getting caught (Dee and Jacob 2010) (see also Power 2009; Gullifer and Tyson 2010). These results indicate that at least some of the current transgression may be resulting from inadequate understanding of the rules and norms of academic integrity more generally rather than being deliberate transgressions of known rules.

A related line of research shows that undergraduate students often face grey-zone situations where acting with integrity requires context-sensitive judgement rather than the straightforward application of black and white rules (Childers and Bruton 2016; Goddiksen et al. 2021). Although actions such as deleting deviating datapoints appear to be transgressive, there are cases where they can be seen to be justifiable, or even required, when the specific context is considered in more detail. In such grey-zone situations students may even be encouraged by their teachers to perform behaviours that appear to break the rules (Johansen & Christiansen 2020).

These research findings strongly suggest that academic integrity will not be improved merely by motivating students to follow clear, well-understood rules. In cases with clear rules, students may indeed fail to comply with the rules either wilfully (an ethical deficiency) or because they neutralize transgressions (a motivational deficiency). But they may also fail to comply because, for one reason or another, they do not understand what the rule requires: this is an *epistemic* deficiency – a lack of knowledge or understanding. Moreover, since not all cases are covered by clear rules, students often face difficult grey-zone situations where acting with integrity requires them to be able to take the context of a given action into account and understand how the rules are to be applied in that particular case.

While investigations such as those mentioned above have added an important dimension to our understanding of academic integrity for undergraduate and other students at university level, only a limited amount of research into the epistemic dimension of academic integrity has been performed at the upper secondary level (with McCabe 1999 and Chu et al. 2020 as exceptions). At this level, the research has sought mainly to survey students’ transgressions of academic rules and norms, and although some research has examined student motives (Sisti 2007; Nora and Zhang 2010; Bacha et al. 2012), we know very little about how the students understand central concepts, and how they apply these concepts in real-life situations. It is our belief that, to promote academic integrity effectively, we need to understand the epistemic complexity surrounding it. Thus, we need to know not only whether students understand key concepts and basic requirements, but also whether they can identify and handle grey-zone situations. In the absence of this information it will be difficult to design teaching materials and other interventions that target the students’ needs.

To address these issues we report from a survey of 1724 upper secondary students at 51 institutions in 6 European countries. The survey looked at student uncertainties, beliefs and practices relating to academic integrity. It focused on integrity issues that arise in the following three main dimensions:

- The use of texts written by others in one’s own work (citation and plagiarism)
- Collaboration with others, assigning authorship and getting help (collaboration and authorship)
- Collection, analysis and presentation of data

The aim of this paper is to identify misconceptions and uncertainties about these three dimensions of academic integrity. We also investigate whether students’ uncertainties are affected by the level of training they have received. Finally, we examine whether students’ levels of self-reported knowledge and uncertainty about the three main areas of academic integrity, academic training in integrity, and perception of peer behaviour, correlate with their engagement in questionable academic practices (QAPs) such as receiving unauthorised help or copying shorter passages from others’ texts without marking them as quotes. We concentrate on the patterns that emerge from the sample as a whole. Differences between the 6 countries and the 51 institutions included in the study fall outside our main focus, but towards the end of the paper we will highlight contrasts between the 6 countries, and summarise possible substantial national differences.

## Methods and materials

### Planning of data collection

The survey was performed as part of the H2020 project INTEGRITY (https://h2020integrity.eu), which aimed to explore academic integrity issues among European students from upper secondary to PhD level. This paper will focus solely on data on the upper secondary students (see Goddiksen et al. 2021 for a report on a qualitative study done as a preparation for the present study).

Initially the survey included upper secondary students from 9 countries: Denmark, Ireland, Lithuania, Portugal, Slovenia, Switzerland, Germany, the Netherlands, and Hungary. In Switzerland we also distinguished between the French and the German speaking regions. However, due partly to the COVID-19 pandemic we only reached the pre-set lower limit of approximately 200 student participants per country in the first 5 of the countries listed above and the French speaking part of Switzerland. Therefore, in the following, the recruitment procedure is only described for these 6 countries (for simplicity, we refer here to the French speaking part of Switzerland as a country).

An institution-level sampling design was employed in all of the countries. A complete list of upper secondary institutions was compiled, and until our target of approximately 200 participants was met, more institutions were randomly drawn and invited to participate. On this common basis, the routes of recruitment varied somewhat across the countries. In Denmark, Ireland and Lithuania only institutions from the random draw were invited. In the remaining 3 countries other recruitment procedures were followed. In the French speaking part of Switzerland, a total population sampling was carried out, so that all 34 institutions in that part of the country were invited. In Portugal, only 3 of the 23 institutions that were randomly drawn agreed to participate, but after this 2 additional institutions were recruited through personal contacts. In Slovenia, a random recruitment procedure was initially followed, but as only 2 institutions volunteered, a recruitment strategy primarily based on personal contacts was adopted.

Once an institution had agreed to take part all, or a relevant sub-set, of their students were invited to participate in the survey. They were invited either through e-mail or they were invited in the course of classes or seminars, by researchers from the INTEGRITY project who visited the institutions. For details, see Additional file 1.

Depending on the structure of the national educational system, in some countries the aim was to recruit only students above the age of 18, while in other countries students from all age groups were recruited. Ethical approval for the study was secured before data collection began (see Additional file 3).

### Data collection and participants

Data were collected from February 2020 to December 2020. As mentioned 3 of the 9 countries originally included in the survey as well as the German speaking part of Switzerland did not reach the pre-set threshold of 200 participants and were therefore excluded (Germany *n =* 17, Hungary *n =* 60, the Netherlands *n =* 60, and German speaking part of Switzerland *n =* 75). In the remaining 6 countries a total of 1724 participants (response percentage 12%) completed the questionnaire (see Table 1).

**Table 1:** Overview of upper secondary institutions and students included in the study

	Upper secondary institutions			Upper secondary students			
	Total in country	Total invited	Total institutions agreeing (participation)^a^	Total in country^b^	Total invited to respond	Total participants	Response percentage
Denmark	190	30	7(7)	86,783	1170	389	33%
Ireland	724	39	13(13)	392,267	932	292	31%
Lithuania	401	112	109(34)	88,864	8561	215	3%
Portugal	704	23	5(5)	399,386	250	218	88%
Slovenia	149	12	7(7)	72,738	1100	250	23%
Switzerland^c^	34	34	19(19)	25,000	2860	360	13%
Total	2202	250	161(85)	1,019,221	14,873	1724	12%

^a^Numbers in parenthesis are counts of schools where at least one completed the questionnaire

^b^Estimates of the 2019/20 population based on publicly available statistics

^c^Only the French speaking part of Switzerland included

Participants were asked which gender they primarily identified with (response options were ‘female’, ‘male’, ‘none of the above’ and ‘prefer not to answer’). A comparison of the distributions of sex and gender identity in the sample and the background population of upper secondary students is shown in Table 2.

**Table 2:** Sex and gender identity in the sample and the background population of upper secondary students (*n =* 1724)

	Distribution in countries among upper secondary students^a^		Distribution in sample			
	Female	Male	Female	Male	None of the above	Prefer not to answer
Denmark	61%	39%	60%	31%	3%	6%
Ireland	50%	50%	58%	38%	1%	3%
Lithuania	51%	49%	58%	28%	2%	12%
Portugal	50%	50%	46%	35%	3%	16%
Slovenia	49%	51%	65%	29%	2%	4%
Switzerland^b^	58%	42%	60%	29%	3%	8%

^a^Estimates of the 2019/20 population based on publicly available statistics

^b^Only French speaking part

Of the 1724 responses, 70 were from institutions where 4 or fewer participants completed the questionnaire. To enable mixed effects analysis at the institutional level, these 70 observations were removed. Following this, the final sample included in the analysis in this study consisted of 1654 participants from 51 institutions. For details, see Table 3.

**Table 3:** Descriptive details regarding the 51 institutions included in analysis – per country and total

	Number of institutions included in study	Average, minimum, maximum, and total number of participants in the institutions			
		Average	Minimum	Maximum	Total participants
Denmark	5	76.8	7	110	384
Ireland	10	29	7	56	290
Lithuania	12	14	5	53	168
Portugal	5	43.6	9	88	218
Slovenia	3	79.7	19	189	239
Switzerland	16	22.2	5	84	355
Total	51	32.4	5	189	1654

Finally, turning to the age distribution in the sample: 452 participants were below the age of 18, 833 were 18 or 19 years, and 369 were 20 years or more (see Table 1, Additional file 2).

## Materials and methods

The design of the questionnaire was based on an explorative interview study of 72 students (18 upper secondary, 18 Bachelor’s and 36 PhD) from Hungary, Denmark and Ireland (see Goddiksen et al. 2021 for details). Several versions of the questionnaire were developed to target student populations at the different educational levels involved in the INTEGRITY project. The questionnaires were then translated into the dominant languages in each of the 9 participating countries and integrated into an anonymous online survey using the platform SurveyXact ver. 12.9 (https://www.surveyxact.com/). Additional file 4 gives further details of the development, pilot testing and translation process. Here we will focus on the parts of the questionnaire specifically addressing upper secondary students.

The questionnaire was dynamic, with the questions presented to a participant sometimes depending on their answers to previous questions. For this reason some participants encountered different, or fewer, questions than others and total numbers of participants are not the same for all questions.

The questions reported in the present study can be divided into seven main categories. We shall now based on these describe the questions and response options, and explain how we developed the measures used in the analysis.

1. Background demographic. Basic demographic questions about age, gender identity, country and name of educational institution. Descriptive details of these were presented above.
2. Uncertainty. For each dimension (i.e. citation and plagiarism, collaboration and authorship, and data collection and analysis) we developed single-item indicators probing about the number of times the participants had had doubts about an issue in the relevant dimension over the past 12 months. Response options were ‘no’, ‘yes, once’, ‘yes, a few times’, ‘yes, many times’, and ‘not applicable’. Participants were presented with the dimension-specific question only if they had confirmed earlier in the questionnaire that their work at upper secondary level had involved these dimensions.
3. Understanding of basic concepts and grey zones. Two types of question were asked in this section. First, the participants were presented with four paraphrases of the same short text and asked, about each, whether the paraphrase was acceptable (on a five-point scale from ‘completely unacceptable’ to ‘complete acceptable’). This design, which followed Roig (1997), aimed to evaluate the participants’ understanding of plagiarism and good citation practice. Second, the participants were asked three sets of questions – one set for each integrity dimension. These probed the students’ understanding of the rules, and whether they could identify grey-zone situations where there are no clear rules. For each question, participants were asked to indicate whether the action described was in contravention of the official rules and regulations applying to them. The response options were ‘yes, it is a serious violation’, ‘yes, but it is not a serious violation’, ‘no, it is not against the rules’, ‘the rules are unclear’, ‘it depends on the situation’, and ‘don’t know’.
4. Academic integrity training. Participants were asked two questions about the training they had received on academic integrity. First, they were asked whether they had taken one or more courses, lectures or e-sessions on any of the three dimensions of academic integrity covered in the survey (we call these *dedicated* courses). Second, they were asked if they had received other forms of training in other courses that were not dedicated to academic integrity, or through teacher feedback on assignments (i.e. during their attendance on *non-dedicated* courses), or through their engagement with non-teachers, including classmates and friends and family. Participants were allowed to give multiple responses.
  After the data had been collected an ambiguity was discovered in the translation into French of the answer options to the first of these questions. This made it difficult to distinguish between the options ‘Yes, one or more dedicated courses’ and ‘Yes, one or more lectures’ in the French version of the questionnaire. Consequently, we merged the two answer options in all of the analyses.
5. Peer perceptions. Participants’ perceptions of questionable behaviour among their peers is a latent construct based on four questions that were designed to reveal what questionable actions the students believed their classmates had engaged in. The actions were: ‘delete data from an experiment only because it somehow seems wrong’, ‘give a misleading or dubious interpretation of texts, works of art or interview data in order to achieve results the teacher will accept’, ‘copy shorter passages from other sources into their own texts without marking them as quotes’, and ‘receive help from other students or family members on assignments they were supposed to complete on their own’. For all four questions, response options were on a five-point scale, ranging from ‘fully agree’ to ‘fully disagree’, and a ‘don’t know’ option in addition (all participants who responded ‘don’t know’ once or more were removed in the evaluation of the construct, as described below). The internal consistency of this construct was acceptable, as the ordinal alpha coefficient – which is the recommended coefficient for binary and ordered items (Gadermann et al. 2012) – was 0.69. We further examined the measurement invariance (also referred to as measurement equivalence) of this latent construct across the included countries, as this is necessary if accurate and meaningful comparison across cultures are to be made (Steenkamp and Baumgartner 1998). There are different levels of measurement invariance. The lowest is configural invariance, which requires that the factor structure is similar across groups (which, in this case, were countries). This is followed by metric invariance, where it is required that the factor loadings between the manifest variables and the latent variable are similar. Finally, there is scalar invariance, where the intercepts of the manifest variables also are required to be similar across groups (e.g. Steenkamp and Baumgartner 1998; Davidov et al. 2014). We aimed to establish metric invariance as a minimum. At this level of invariance, factor variances and covariances cannot be attributed to measurement error owing to country-specific differences in the scale properties. So we could be sure that it was ‘true’ differences in the construct (in this case, peer perceptions) that explained the questionable behaviour (Gregorich 2006). Scalar invariance is a requirement for making meaningful and unbiased comparisons of group means of the latent construct. It was not strictly a requirement in the present study to obtain this level of invariance, since we were not aiming to compare country means. We used the guidelines employed by Davidov (2009) to assess measurement invariance. This involves the execution of a series of single confirmatory factor analyses (CFA) and multi-group CFA using a bottom-up test strategy. The strategy starts with the weakest level of invariance, after which restrictions are gradually imposed on the measurement model until the scalar level is tested. The following global fit measures were used to identify well-fitting models (acceptable values are given in parentheses): the root mean square error of approximation (RMSEA) (acceptable value: < 0.05), the standardised root mean squared residual (SRMR) (acceptable value: < 0.08), and the comparative fit index (CFI) (acceptable value: > 0.95). The assessment of invariance was carried out using the *sem* command in Stata. Turning to our assessment of invariance: we did not establish scalar invariance for the construct (CFI 0.87/RMSEA 0.092 (90%CI: 0.073–0.112)/SRMR 0.062/ Chi^2^ 124.52(46)). However, we identified the construct as having satisfactory metric invariance (CFI 1.0/RMSEA 0.00 (90%CI: 0.00–0.047)/SRMR 0.057/ Chi^2^ 22.84(26)) (see Additional file 7 for details), which, as mentioned above, was sufficient for the analysis presented in this paper. We derived factor scores from the multi-group CFA (using Stata’s *predict* command) so that the peer perception measure could be used in subsequent analysis. Participants who answered ‘don’t know’ to two or more of the four questions making up this measure were not assigned a score (and treated as missing).
6. Self-reported knowledge. This is a latent construct based on six questions. For all three of the dimensions of academic integrity (i.e. citation and plagiarism, collaboration and authorship, and data collection and analysis) the participants were asked to indicate (i) their self-perceived level of knowledge, and (ii) whether they knew how to behave ethically correctly. The response options were set out on a five-point scale ranging from ‘completely disagree’ to ‘completely agree’. In addition, there was a ‘don’t know’ option (all participants that responded ‘don’t know’ once or more were removed in the evaluation of the construct below). The internal consistency of this construct was acceptable, as the ordinal alpha (Gadermann et al. 2012) was 0.80. In a contrast with the peer perception measure described above, however, here we were not able to identify configural or metric equivalence. Further, the fit indices from single-country CFA did not meet the requirements described earlier for good model fit. On the positive side, though, all manifest variables loaded highly on the first factor (see Additional file 8 for details) in all countries. So the measure does tap into self-reported knowledge, but not particularly accurately, and not in the same way across countries. Despite this limitation, we decided to use the construct in the analysis because of its importance to the research questions. We derived factor scores from CFA (using Stata’s *predict* command) so the self-reported knowledge measure could be used in subsequent analysis.
7. Questionable academic practice. Participants were presented with four questionable practices: ‘deleted deviating data points based on a gut feeling that they were inaccurate’, ‘copied shorter passages from other sources into your own text without marking them as quotes’, ‘added students as co-authors of group assignments, even though they did not contribute’, and ‘received help from other students or family members on assignments you were supposed to complete on your own’. For each practice participants were asked whether, and if so, how much, they had engaged in the practice. The response options were ‘no’, ‘yes, once’, ‘yes, a few times’, ‘yes, many times’, ‘I prefer not to answer’, ‘Not applicable, and ‘don’t know’.

The English version of the questionnaire is included as Additional file 5.

### Data analysis

Descriptive statistics were reported for all single-item measures of self-reported knowledge and uncertainty, understanding of basic concepts and grey zones, academic integrity training, and questionable academic practices.

Spearman’s correlation coefficients were used to examine the effects of formal and informal academic training on participants’ level of uncertainty. Mixed effects multivariable logistic regression analyses were conducted to investigate whether the participants’ academic training in integrity and level of self-reported knowledge and uncertainty about the three main areas of academic integrity predicted their engagement in questionable practices. In this analysis, we collapsed the four indicators of questionable practice into binary outcomes, where 1 indicates that the questionable practice has been employed at least once during the past year and 0 that it has not (0 = ‘no’, 1 = ‘yes, once’, ‘yes, a few times’, ‘yes, many times’). The dimension-specific measures of uncertainty were inserted as individual-level continuous predictors when they matched the dimension measured in the outcome variable. Further, two indicators of dedicated academic training (attending lecture/course, and attending e-session) and three indicators of non-dedicated academic training (feedback on written work or assignments, courses not dedicated exclusively to academic training, and discussions with teachers outside regular classes) were inserted as binary predictors. Self-reported knowledge and peer perceptions were inserted as continuous predictors. The following control variables were inserted: gender and age (inserted as a categorical variable). In all analyses, institutional ID was included as random intercept. Descriptive details of the predictor variables used in the mixed effects models are set out in Additional file 2, Table 1. The sample size of the four models varied because participants were removed if they answered ‘I prefer not to answer’, ‘Not applicable, or ‘don’t know’ to the questionable behaviour in question, or if they had responded ‘don’t know’ or ‘not applicable’ to the dimension-specific questions about self-reported knowledge and uncertainty. The mixed effects analyses were run in Stata/MP version 17 using the *melogit* command. We used the backward elimination approach in which predictors were consecutively removed until all of the remaining predictors were statistically significant. Statistically significant effects were identified using Stata’s post-hoc *test* command, with *p* < 0.05 considered significant. For all models, we briefly summarised statistically significant effects, but we focused on characterising the impact of the four main constructs of interest (level of self-reported knowledge, uncertainty, peer perceptions, and academic training in integrity) on questionable behaviour. We characterised the strength and direction of these four constructs by reporting predicted probabilities using Stata’s *margins* command. For self-reported knowledge and peer perceptions, the predicted probabilities were reported at three gradient levels: 1 standard deviation (SD) below the Mean, Mean, and 1 SD above the Mean. The full output from the four regression models are laid out in Additional file 9.

In all of the analyses where statistical tests were conducted, the 0.05 probability level was used as the criterion of statistically significant association. Stata version 17 and SPSS version 28 were used to conduct the analyses.

## Results

### Knowledge and uncertainty

When asked to evaluate their own level of knowledge, a large majority of the participants expressed the belief that they had a good understanding of the standards of good practice that apply to them. In the two dimensions ‘Collection, analysis and presentation of data’ and ‘Working with others and assigning authorship’, 80% and 71%, respectively, agreed or fully agreed that they had a good understanding of the standards. The confidence of the participants was slightly lower for the last dimension ‘Citation and plagiarism’, where only 65% agreed or fully agreed that they had a good understanding (see Table 4).

**Table 4:** Self-reported knowledge of how to behave ethically correctly in relation to three dimensions of academic integrity: ‘I have a good understanding of the official standards of good practice that apply to me in relation to..’

	Fully agree	Agree	Neutral	Disagree	Fully disagree	I don’t know
Citation and plagiarism (*n =* 1654)	22.8%	41.7%	17.8%	7.9%	4.5%	5.4%
Working with others and assigning authorships (*n =* 1551)	23.8%	46.7%	18.8%	5.0%	1.9%	3.7%
Collection, analysis and presentation of data (*n =* 1220)	25.8%	54.3%	13.8%	2.8%	0.5%	2.8%

When the participants were asked if they knew how to behave in an ethically correct manner in relation to the same three dimensions, similar, but slightly higher, levels of agreement were found (see Table 5).

**Table 5:** Self-reported understanding of what is good practice in relation to three dimensions of academic integrity: ‘In general, I know how to behave in an ethically correct manner in relation to..’

	Fully agree	Agree	Neutral	Disagree	Fully disagree	I don’t know
Citation and plagiarism (*n =* 1654)	25.5%	44.1%	16.3%	6.7%	3.0%	4.4%
Working with others and assigning authorships (*n =* 1551)	28.2%	46.4%	16.2%	5.0%	1.4%	2.9%
Collection, analysis and presentation of data (*n =* 1220)	27.6%	51.1%	15.6%	3.0%	0.6%	2.0%

Turning to uncertainty, where each of the three dimensions of academic integrity were concerned, about half of the participants had had doubts about how to act ethically correctly at least once during the past year (see Table 6). The participants reported slightly more uncertainty in connection with the dimension of citation and plagiarism (where 54% had been in doubt at least once) than with the other two dimensions.

**Table 6:** Self-reported levels of uncertainty in relation to three dimensions of academic integrity during the past 12 months: ‘Over the past 12 months, have you been in a situation where you were unsure how to behave in an ethically correct manner in relation to … ’

	Yes, many times	Yes, a few times	Yes, once	No	Not applicable
Citation and plagiarism (*n =* 1654)	5.1%	26.4%	21.9%	41.5%	5.0%
Working with others and assigning authorships (*n =* 1551)	4.7%	23.9%	15.9%	49.6%	5.9%
Collection, analysis and presentation of data (*n =* 1220)	8.7%	27.1%	18.7%	41.5%	4.0%

### Understanding of concepts and grey zones

To obtain a more direct evaluation of their understanding of plagiarism, we presented the participants with a paraphrasing scenario in which a friend wanted to use a paragraph from a textbook in the introduction of an assignment. We asked the participants whether they thought the friend’s actions were acceptable. In using the term ‘acceptable’ we were asking about the participants’ judgement of the case. This gives insight into participants’ perception and understanding of what constitutes acceptable behaviour, and not necessarily their knowledge of the rules. Perceptions of the rules were probed in the following questions.

The four uses of the original text can be characterised as follows (full details in Additional file 5):

- Paraphrase 1: a direct copy without quotation marks and no reference.
- Paraphrase 2: a few insignificant words has been deleted or replaced with synonyms, but still without a reference to the original.
- Paraphrase 3: identical to paraphrase 2, except that a reference to the original had been added.
- Paraphrase 4: a more substantial rewriting than that in 2 with a reference to the original

**Fig. 1:**
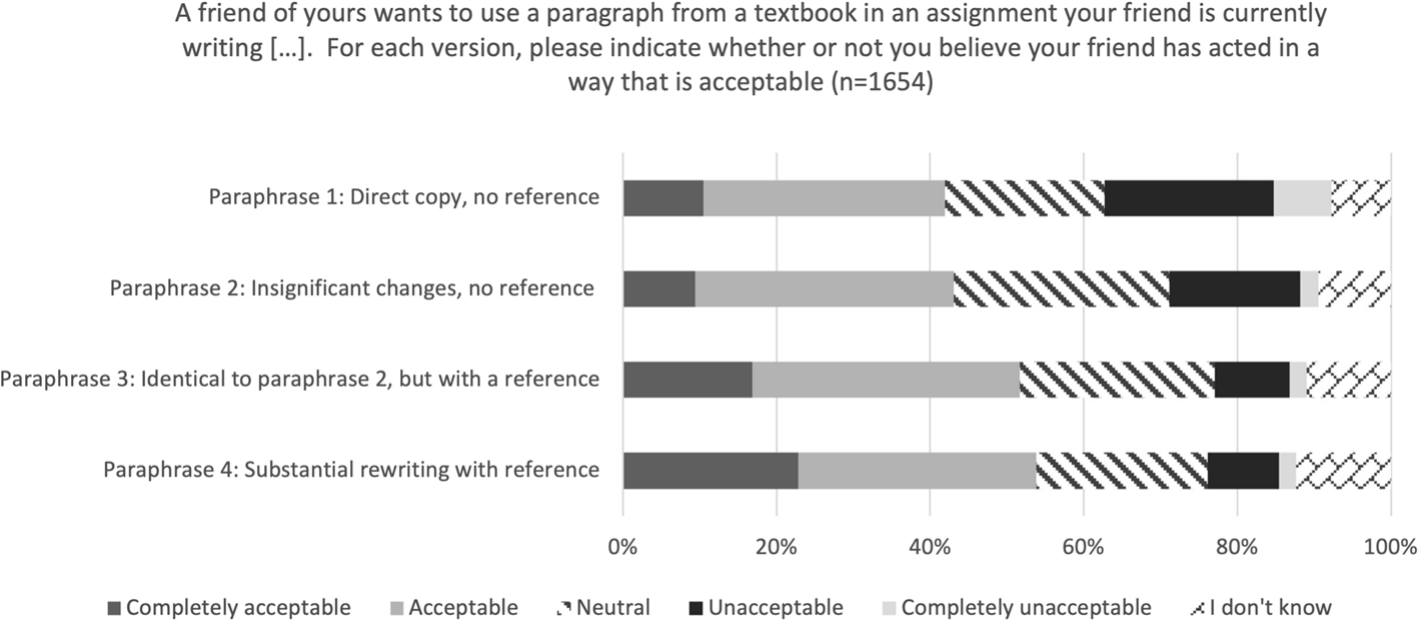
Participants’ perception of the acceptability of various forms of paraphrasing a paragraph from a textbook in an assignment

Paraphrase 1 (which is actually unattributed copying, not paraphrasing) is a clear instance of plagiarism, and we expected students to consider it as an obviously unacceptable thing to do. However, 42% of the participants considered it to be acceptable or completely acceptable (see Fig. 1). The four paraphrases were constructed to appear increasingly acceptable in the sense that they are increasingly removed from verbatim copying and failure to attribute a source. Although it is difficult to point out precisely when a paraphrase is acceptable, we expected students with a robust understanding of plagiarism to be able to see differences in the acceptability of the various scenarios. We observed a difference in the participants’ evaluation of a direct, unattributed copy (Paraphrase 1) and a copy with a few insignificant changes without referring the original source (Paraphrase 2), with more participants (30%) considering the first to be unacceptable or clearly unacceptable than the latter (19%). Similarly, there was a perceived difference between a paraphrase without a reference (Paraphrase 2) and paraphrases with a reference (Paraphrases 3 and 4). There was also a difference between Paraphrases 1 and 2, on the one hand, and 3 and 4, on the other, although the difference is seen in the other end of the spectrum. Here, participants were more likely to consider Paraphrases 3 and 4 acceptable or completely acceptable (52% and 54%, respectively) than Paraphrases 1 and 2. The overall change in participants’ perceptions across the four scenarios was, however, relatively small.

To probe how the participants handled grey-zone situations calling for context-sensitive judgement, we asked them to evaluate whether, in their opinion, various actions contravened the rules applying to them. Four questions were asked for each of the first two dimensions of academic integrity under consideration in the study, and three questions were asked for the third dimension.

**Fig. 2:**
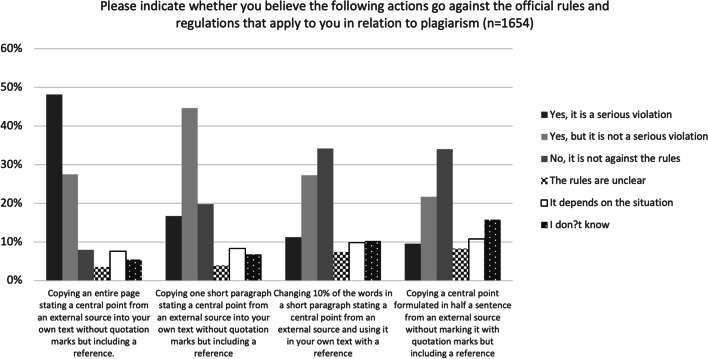
Perceptions of what constitutes a violation of the rules and regulations on plagiarism

Beginning with plagiarism and citation practice, participants were presented with four scenarios representing different degrees of plagiarising ranging from copying an entire page to copying a central point alone (see Fig. 2). It is worth noticing that just under half of the participants (48%) perceived copying an entire page stating a central point without quotation marks as a serious violation. Furthermore, 20% stated that it is not against the rules to copy a short paragraph without quotation marks but including a reference. The participants did seem to recognise, however, that there is a difference between copying an entire page and copying only a short paragraph, as they clearly considered the latter to be a less serious violation or not a violation at all.

Relatively few of the participants used the options ‘The rules are unclear’ or ‘It depends on the situation’. Thus 11% used one of these options for the first question, 12% for the second question, and 17% and 19%, respectively, for the last two question.

**Fig. 3:**
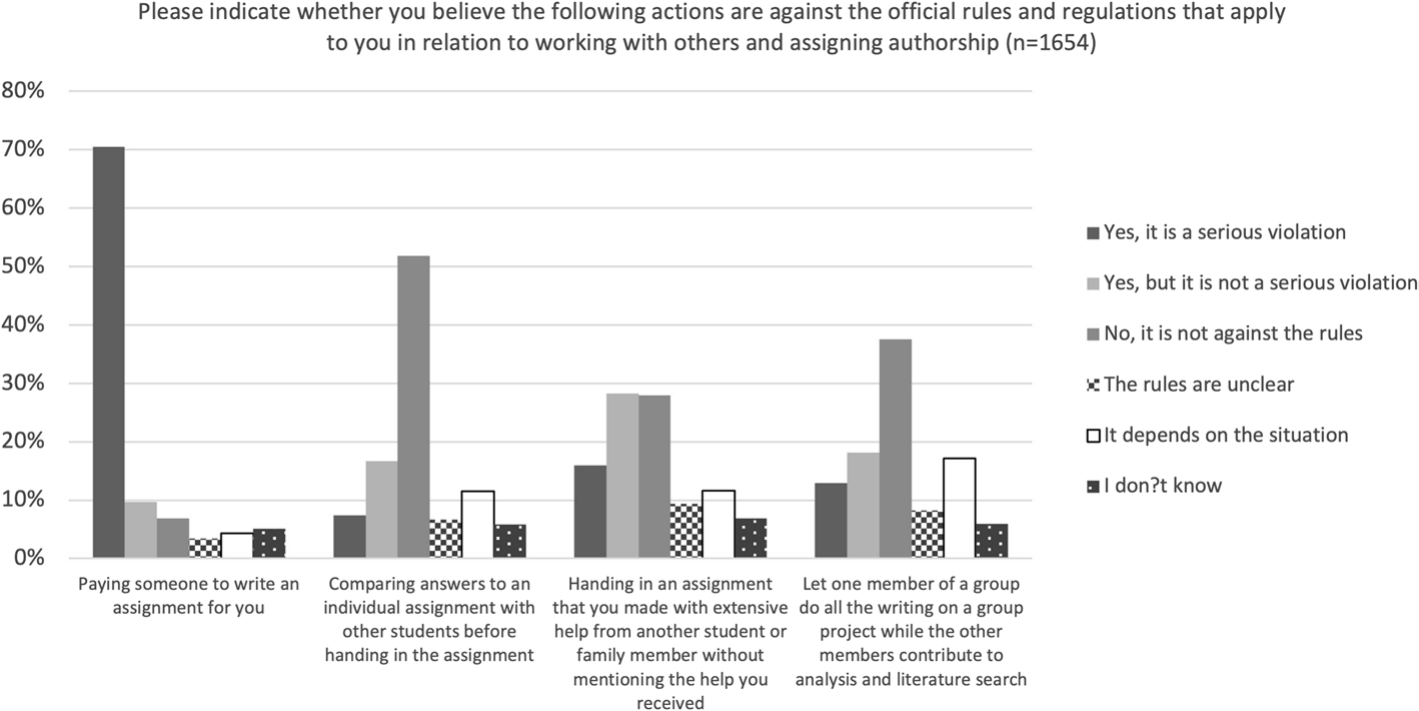
Perceptions of what constitutes a violation of rules and regulations when working with others and assigning authorship

Where collaboration was concerned, the participants were presented with four situations involving aspects of collaboration – namely, buying assignments, comparing answers with others, receiving help from others and free-riding (see Fig. 3 for details). More than 70% of them considered it a serious violation to pay someone to write an assignment for them. They were more divided on the other questions, but again here relatively few used the options ‘The rules are unclear’ or ‘It depends on the situation’. Only 18% of the participants used one of these options for the second question (comparing answers), and respectively 21% and 25% used one of the options for the two last questions (receiving help from others and authorship attribution).

**Fig. 4:**
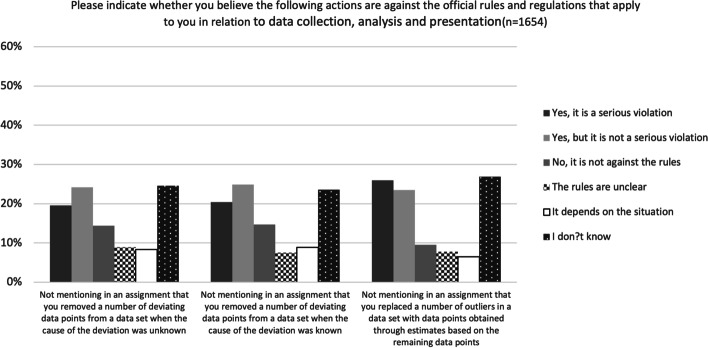
Perceptions of what constitutes a violation of rules and regulations covering the collection, analysis and presentation of data

Finally, the participants were given three questions about different ways of justifying the exclusion of deviating data. The first two probed whether the participants saw a difference between removing deviating data when the cause of the deviation is known and doing so when the cause is unknown. The last question focused on whether the participants regarded the construction of data and simple filtration of data differently. For all three questions a significant minority (24–27%) chose the option ‘I don’t know’, while a smaller percentage (14–17%) chose the options ‘The rules are unclear’ or ‘It depends on the situation’ (Fig. 4). Although the participants were divided in their answers to all three questions, there were only small changes in the distribution of answers.

### Academic integrity training and its impact on uncertainty

Approximately one third (34%) of the participants reported that they had attended dedicated training on academic integrity; either course/lecture or e-learning session (Table 7). The majority of them had received some form of training delivered in other ways (Table 8), *either* through comments from instructors on written work and/or assignments (44%), courses not dedicated to academic integrity (20%) or discussions with teachers outside classes (17%) (pooled share of those who had engaged with one or more of these forms of training: 60%), *or* in informal settings such as discussions with friends or other students (46%).

**Table 7:** Engagement with dedicated training on academic integrity (*n =* 1654)

Have you taken courses on rules and/or ethically correct behaviour in relation to the themes introduced above during your current or previous studies?	%
Yes, one or more dedicated courses/lectures	29.9
Yes, one or more dedicated e-learning sessions	5.1
No	66.1

Shares sum to more than 100% because both of the ‘yes’ options were possible

**Table 8:** Engagement with other forms of training in academic integrity (*n =* 1654)

Have you learned about rules and/or ethically correct behaviour in relation to the themes introduced above through any other method?	%
Yes, through supervisors/teachers in other courses that commented on my written work or assignments	43.6
Yes, through courses not dedicated exclusively to such issues	20.1
Yes, through discussions with teachers outside regular classes	16.8
Yes, through discussions with fellow students	26.8
Yes, through self-study	18.3
Yes, through discussions with friends and family outside my institution	27.3
Yes, other	8.8
No	13.1
I don’t know	11.1

Shares sum to more than 100% because multiple answers were possible for the eight first options. The ‘I don’t know’ option was a single choice option

To understand the impact of training we investigated the relationship between the participants’ level of uncertainty in each of the three main integrity dimensions (citation and plagiarism, collaboration and authorship, and collection and analysis of data) and all of the types of training shown in Tables 7 and 8 that involved teachers (Table 9).

**Table 9:**
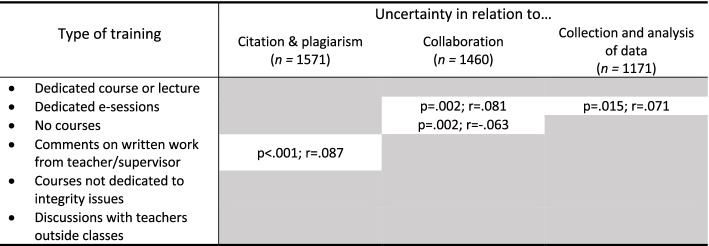
Associations between informal and formal training received, and levels of uncertainty regarding three dimensions of academic integrity

Spearman’s correlations coefficients are reported in the table. Grey shaded cells indicate that no correlation was detected using a 5% significance level (i.e. *p* > .05)

The results show that, in most cases, there is no association at all between type of training and uncertainty. In the four cases where there is a statistically significant association, the strength of the correlation between training and uncertainty is rather weak and will probably have very little real impact (Spearman’s correlation coefficients < 0.100).

### Questionable Academic Practices (QAPs)

We asked the participants if they had engaged in practices such as copying shorter passages or receiving help from friends or family on assignments they were expected to complete on their own (Table 10). These actions include minor violations of the rules (e.g., copying shorter passages) and infringements whose exact status depends to some extent on the context (e.g., deleting deviating data points).

**Table 10:** Self-reported engagement in questionable academic practices among upper secondary students (*n =* 1654)

Questionable practice	Prevalence						
During your high school education have you...	Yes, many times	Yes, a few times	Yes, once	No	Not appli-cable	I prefer not to answer	I don’t know
… copied shorter passages from other sources into your own text without marking them as quotes	10.6%	26.1%	15.7%	34.8%	1.3%	2.4%	9.1%
… added students as co-authors of group assignments even though they did not contribute	13.3%	24.9%	14.1%	34.8%	1.7%	2.7%	8.4%
… received help from other students or family members on assignments you were supposed to complete on your own	17.5%	41.1%	13.8%	17.6%	1.3%	2.5%	6.3%
… deleted deviating data points based on a gut feeling that they were inaccurate	4.7%	18.7%	16.9%	41.8%	2.5%	2.2%	13.2%

Receiving help from other students or family was a very common QAP, with 72% of the participants admitting to having done it at least once. A slim majority also admitted to having copied shorter passages at least once (52%), and to having given undeserved co-authorship to other students (52%). Fewer participants (40%) admitted engagement in QAPs connected with data collection and analysis. A high share of the participants (78%) said they had engaged in at least one of the four QAPs at least once.

Results of the multivariate mixed effects regression analyses designed to identify the factors that explain the four QAPs are set out in Table 11.

**Table 11:** Overview of individual level explanatory factors predicting the analysed questionable practices (results from multivariate mixed effects analyses)

Questionable practice (QAP)	Significant association with
Copied shorter passages from other sources into your own text without marking them as quotes (*n =* 1218)	• Uncertainty concerning citation practices • Academic training (courses not dedicated to integrity issues) • Country • Perception of peer behaviour
Added students as co-authors of group assignments even though they did not contribute (*n =* 1139)	• Gender • Country • Perception of peer behaviour
Received help from other students or family members on assignments you were supposed to complete on your own (*n =* 1168)	• Uncertainty concerning collaboration • Academic training (discussions with teachers outside regular classes) • Perception of peer behaviour
Deleted deviating data points based on a gut feeling that they were inaccurate (*n =* 902)	• Uncertainty concerning data • Self-reported knowledge • Perception of peer behaviour

Here we focus on the impacts of the four factors that are of interest in this paper, namely: uncertainty, self-reported level of knowledge, academic training, and peer perception. The predicted probabilities for the first three of these factors are given in Tables 12, 13 and 14 (when their effects were statistically significant) and those for peer perception are given in Table 15.

**Table 12:** Predicted probabilities for QAP ‘Copied shorter passages from other sources into your own text without marking them as quotes’. For each level of uncertainty and academic training the probability that participants with this level of uncertainty/training engage in this QAP is indicated (*n =* 1218)

Uncertainty concerning citation practice^a^	Probability of engaging in the QAP
Yes, many times	0.68
Yes, a few times	0.65
Yes, once	0.61
No	0.57
Academic training (Courses not dedicated to integrity issues)^b^	Probability of engaging in the QAP
Yes	0.55
No	0.63

^a^Based on the question: ‘Over the past 12 months, have you been in a situation where you were unsure how to behave in an ethically correct manner in relation to citation and plagiarism?’ The reported probability indicates that the participant carried out the QAP at least once during the past 12 months

^b^Based on participants selecting the option ‘Courses not dedicated to integrity issues’ when asked: ‘Have you learned about rules and/or ethically correct behaviour in relation to the themes introduced above through any other method?‘

**Table 13:** Predicted probabilities for QAP ‘Received help from other students or family members on assignments you were supposed to complete on your own’. For each level of uncertainty and academic training the probability that participants with this level of uncertainty/training engage in this QAP is indicated (*n =* 1168)

Uncertainty concerning collaboration^a^	Probability of engaging in the QAP
Yes, many times	0.86
Yes, a few times	0.84
Yes, once	0.82
No	0.79
Academic training (discussions with teachers outside regular classes)^b^	Probability of engaging in the QAP
Yes	0.87
No	0.80

^a^Based on the question: ‘Over the past 12 months, have you been in a situation where you were unsure how to behave in an ethically correct manner in relation to working with others and assigning authorship?’

^b^Based on participants selecting the option ‘discussions with teachers outside regular classes’ when asked: ‘Have you learned about rules and/or ethically correct behaviour in relation to the themes introduced above through any other method?’

**Table 14:** Predicted probabilities for QAP ‘Deleted deviating data points based on a gut feeling that they were inaccurate’. For each level of uncertainty and stated knowledge the probability that participants with this level of uncertainty/knowledge engage in this QAP is indicated (*n =* 902)

Uncertainty concerning data^a^	Probability of engaging in the QAP
Yes, many times	0.69
Yes, a few times	0.61
Yes, once	0.53
No	0.44
Self-reported knowledge	Probability of engaging in the QAP
1 SD below Mean	0.57
Mean	0.53
1 SD above Mean	0.48

^a^Based on the question: ‘Over the past 12 months, have you been in a situation where you were unsure how to behave in an ethically correct manner in relation to collection, analysis and presentation of data?’

**Table 15:** Predicted probabilities depending on perception of peer behaviour. For each QAP and each level of perceived peer engagement in QAPs the predicted probability that participants with this peer perception will engage in the QAP is reported at three gradient levels

Perception of peer engagement in QAP	Copied shorter passages from other sources into your own text without marking them as quotes (*n =* 1218)	Added students as co-authors of group assignments even though they did not contribute (*n =* 1139)	Received help from other students or family members on assignments you were supposed to complete on your own (*n =* 1168)	Deleted deviating data points based on a gut feeling that they were inaccurate (*n =* 902)
1 SD below Mean	0.47	0.54	0.77	0.36
Mean	0.62	0.61	0.82	0.54
1 SD above Mean	0.75	0.68	0.85	0.71

Tables 12, 13 and 14 demonstrate a clear trend. Uncertainty increased the participants’ likelihood of engaging in QAP (for three of the four QAPs examined) and knowledge reduced QAP (in one case). The effect of academic training was more ambiguous.

For all four of the QAPs, the participants’ own behaviour was correlated with their perceptions of their peers’ behaviour. Participants who believed that a given questionable practice was common among their peers were more likely to engage in it themselves (Table 15).

### Differences between countries

All of the descriptive data reported above in aggregated form is reported with stratification by country in Additional file 6. In connection with most of the questions there are differences between countries. For self-reported level of uncertainty, for instance, the number of participants who had experienced uncertainty at least once in relation to citation and plagiarism ranges from 44% in Lithuania to 65% in Switzerland, and the number of participants who had experienced uncertainty at least once in relation to collaboration ranges from 32% in Slovenia to 58% in Ireland. As regards level of academic integrity training, the number of participants who reported that they had not received dedicated lectures or similar ranges from 43% in Denmark to 87% in Slovenia. Country was a significant factor for two of the four QAPs included in the survey (‘Copied shorter passages…’, and ‘Added students as co-authors’: see Table 11), and here we do see a trend as participants from the two countries with the most formal training, Denmark and the French speaking part of Switzerland, appeared to be slightly less likely to engage in questionable practices. This pattern however breaks as participants from Denmark most frequently included undeserved co-authorship (77%) and Swiss students were relatively prone to delete data without justification (53%).

In sum, although several differences between the countries can be pointed to, it is difficult to identify clear and consistent trends in the data. We cannot say that one country (or a group of countries) deviates from the others in a clear and consistent way.

## Discussion and conclusions

The upper secondary students in the survey generally believed they had a good understanding of the rules and knew how to behave with academic integrity. Our results show, however, that large numbers of the participants struggled to give correct answers to questions about best practice in concrete situations. Asked to evaluate the ethical status of the four paraphrases we presented them with (Fig. 1), only a minority (30%) understood that direct copy is unacceptable, and only a small minority seemed to understand that rewriting and referencing would improve matters and lead to a different ethical evaluation of the paraphrase. This indicates that the participants lacked practical understanding of how to incorporate texts written by others in their own texts in an ethically acceptable way.

In relatively clear-cut situations, where one would expect upper secondary students to know how to act, many of the participants gave answers that did not accord with best practice. For example, less than half considered plagiarising an entire page to be a serious violation of the rules. Similarly, very few were able to identify grey-zone situations that require context-sensitive judgements. Context will typically determine, for instance, whether comparing answers in an individual assignment is permitted, but less than 20% of the participants showed that they recognised this by answering ‘the rules are unclear’ or ‘it depends on the situation’ (Figs. 2, 3 and 4).

Finally, it is also worth noting that the participants seemed to have very little understanding of how to handle deviating data (Fig. 4). Although students at secondary level may not be required to possess this understanding, deficits in it could be a significant problem for tertiary institutions requiring appropriate data practice.

In short, the participants’ belief that they have a robust understanding of the rules and ethical requirements defining academic integrity was largely a misconception. Rather, to borrow an expression from Kruger and Dunning (1999), the participants seemed to be both unskilled and unaware of it.

This lack of knowledge is understandable considering the relatively low level of dedicated training the students reported (Tables 7 and 8). Although it is important to stress that we did not have access to data on actual participation in training, but only to the participants’ recollections, the shares seem low. Almost two-thirds of the participants did not recall having taken courses, or heard lectures, dedicated to the topic. On a more positive note, a majority recalled having learned about academic integrity in other ways, including through comments from teachers and discussions with fellow students. Surprisingly, teacher-delivered training did not seem to have had a sizeable effect on the participants’ levels of uncertainty. Although two forms of non-dedicated training had an effect on the participants’ propensity to engage in questionable practices of the kind we examined, the effects were small and ambiguous (in one case training increased the propensity and in another it decreased it). It is a concern that current teacher-delivered training (dedicated and non-dedicated) apparently does not lower the students’ levels of questionable behaviour and only has a very small effect if any on their levels of uncertainty. This shows that there is a need to revise and rethink the way academic integrity is taught at the upper secondary level.

It is also worth noting that the participants’ levels of uncertainty correlated with their propensity to engage in questionable behaviours – at least, in three of the four QAPs we examined. Uncertainty of the kind we were investigating can be interpreted in two rather different ways. In connection with grey-zone situations, where you must make context-sensitive judgements, uncertainty might be better than overconfidence, but in relatively clear-cut situations, such as plagiarising large portions of text, students should not be in doubt about how to act and the uncertainty is an indication of a lack of knowledge and training. Since the participants in our survey seemed to have a real knowledge deficit where the basic rules and requirements of academic integrity are concerned, it is fair to conclude that at least part of their uncertainty is of the second type, and is thus an indication that they lack knowledge.

Among the four factors we investigated, participants’ propensity to engage in questionable behaviour was most clearly correlated with their perceptions of peer behaviour (Table 15). This effect is well-known in university level students (McCabe and Treviño 1993; McCabe et al. 2001; O’Fallon and Butterfield 2012) and has also been shown to be present at upper secondary level (Stephens and Gehlbach 2007). Our results clearly confirm this connection. Although the direction of causality can be discussed (do students break the rules because they believe their peers break the rules, or do they believe their peers break the rules because they do so themselves?) the existence of a robust association between beliefs about social context and own actions has clear educational implications (see Griebeler 2018 for a model of the effect). In particular it shows that the problem of norm transgression cannot be reduced simply to questions about individual ethics or knowledge.

Overall, our results offer a new perspective on the academic integrity of upper secondary students, one that differs from the explanations generally found in the more traditional parts of the literature. The misconceptions and lack of knowledge we have identified can arguably lead to situations where students accidentally, rather than intentionally, transgress a rule simply because they do not understand what it requires of them. This has clear implications for the way the problem of compliance needs to be addressed. If students break the rules out of ignorance, increased policing and harsher punishments (e.g., Kessler 2003) may not be the most productive response. Similarly, although ethical character building through honour codes or similar may be valuable for a variety of reasons, it is probably not enough to solve the problem of academic integrity. Students need to understand what acting with academic integrity means before they can make ethically informed choices about how to act. The students in our study clearly lacked this understanding.

Of the 10 principles to reduce classroom cheating developed by McCabe and Pavela 1997 (see also McCabe et al. 2001), the very first asks teachers to communicate their expectations about appropriate behaviour and cheating clearly. The results presented above underline that this is indeed crucial, at least as regards the academic integrity of upper secondary students. If less than half of the students at this point in their education view plagiarising an entire page as a serious violation of the rules, something has clearly gone wrong in the communication of expectations.

Fortunately, more training aimed at providing students with the appropriate knowledge has been developed during the last decade. The natural experiment by Dee and Jacob (2010) described above illustrates one form this training can take, but a number of similar online tools aimed at improving university students’ understanding and raising their awareness of academic integrity issues have recently been developed (e.g., Curtis et al. 2013; Cronan et al. 2017; Stephens et al. 2021). Our results suggest that it might be fruitful to develop similar tools for the upper secondary level.

To return to the comparison of the six countries we examined, we did identify national differences here in the participants’ behaviour and conceptions of academic integrity. We had expected to see clear patterns emerge – either in the sense that participants from a given country would have a clear academic integrity profile (e.g., as a group with very strong competences) or because students from countries with similar school systems would behave in similar ways. It is difficult, however, to see any clear patterns in the data comparing countries. Although this is a null result, it is nonetheless interesting to discover that national background had no tangible influence on the participants’ understanding and conceptions of academic integrity. Nor did the specific steps regarding integrity training taken in the individual countries seem to have had an appreciable positive influence on the participants.

Several limitations of the study need to be noted. Different recruitment methods were used depending on local conditions, some of which did not involve random selection of the institutions to be invited. The participants also completed the questionnaire under different conditions, ranging from in-class, as part of a seminar, to at-home, in response to an e-mail invitation (we note that recruitment and data collection took place during the COVID-19 lockdown, which explains some of the difficulties we encountered). These recruitment differences led to large differences in response rates. In countries where we relied primarily on e-mail invitations (e.g., Lithuania) the response rate was low, while it was high in countries relying primarily on in-class recruitment (e.g., Portugal). Further, the participants were drawn from very few institutions in Denmark, Portugal and Slovenia, which may have affected the representativeness of the findings in these countries. The extent to which these data limitations affected our results, through nonresponse bias, for example, is difficult to assess. However, we can note that the sex and gender identity misrepresentation, with male identifying participants being slightly under-represented in the data (see Table 2), presumably had a modest impact on the study findings, as gender was associated with questionable behaviour to a very limited extent in the multivariate analyses. While response rates are problematic only if there is non-response bias (Davern 2013), the very low response rate in Lithuania is a clear cause of concern. Despite the elevated risk of non-response bias in the Lithuanian data, we chose to retain them because Lithuania was the only North-East European country represented in the study. It is worth noting that our assessment of invariance for the peer perception measure indicated that the construct worked well in Lithuania (Additional file 7). So the construct quality, at least, in the Lithuanian sample is on par with that for the other study countries. Finally, it is a limitation of the study that we cannot claim that all of the main theoretical constructs used in it have equivalent meaning across the countries. In particular, uncertainty is a single-item indicator for which established methods and guidelines to detect measurement invariance cannot be carried out (Steenkamp and Baumgartner 1998; Gregorich 2006; Davidov et al. 2014). Moreover, for self-reported knowledge we did not identify measurement invariance even at the weakest (factorial) level. For this reason, the analyses where these measures were included should be treated with caution. In the worst case, the statistically significant or non-significant effects from these two variables could be ascribed to measurement error.

It should also be acknowledged here that the comparison between countries is further challenged by the pragmatic choice, in some countries, to recruit mainly students in their senior year, while a broader recruitment profile was achieved in other countries.

## Supplementary Information

**Additional file 1.** Details on recruitment procedures

**Additional file 2.** Descriptive statistics for the predictor variables

**Additional file 3.** Ethical approval

**Additional file 4.** Development, testing and translation of questionnaire

**Additional file 5.** English version of the questionnaire

**Additional file 6.** Data stratified by country

**Additional file 7.** Assessment of measurement invariance of peer perception

**Additional file 8.** Assessment of measurement invariance of self-reported knowledge

**Additional file 9.** Output from the four regression models

## Data Availability

The survey instrument is available as Additional file 5. All data is available online at 10.17894/ucph.66293057-46c5-4d02-a116-dfd597ce5a78.

## Abbreviations

CFA: Confirmatory Factor Analyses
CFI: Comparative Fit Index
QAP: Questionable academic practice
RMSEA: Root Mean Square Error of Approximation
SD: Standard Deviation
SRMR: Root Mean Squared Residual
