# Supplementary material for: Lack of ethics or lack of knowledge? European upper secondary students’ doubts and misconceptions about integrity issues
Source: Int J Educ Integr. 2022 Aug 11;18(1):20. doi: 10.1007/s40979-022-00113-0 (PMC9365441; doi:10.1007/s40979-022-00113-0)
Supplement: Supplementary file 1 — Additional file 1. Details on recruitment procedures [file 40979_2022_113_MOESM1_ESM.pdf]

Additional file 1: Details on recruitment procedures

Denmark: Only students above the age of 18 were included in the survey. To comply with this restriction, we recruit only students who were in their senior year. Institutions that were chosen through the random draw was approached and given the choice to either send out an e-mail invitation including a link to the online survey to all senior year students or to host seminars where researchers from University of Copenhagen could teach their students about academic integrity after the students had filled out the questionnaire in class. If institutions agreed to host seminars, all senior classes would be invited. Students below the age of 18 were allowed to participate in the seminars, but were not invited to the survey.

Portugal: Initially, randomly selected institutions were approached and invited to participate in the survey. Institutions that accepted the invitation were asked if a member of the Portuguese team could visit in-person to provide a quick briefing about the INTEGRITY project and about the survey to the students before they answered the survey. Due to the COVID-19 pandemic only one such visit was carried out. Two other institutions had accepted to participate. Here, teachers introduced the project based on a PowerPoint presentation developed by the Portuguese team before the students answered the survey. Due to COVID-19 the initial plan to recruit randomly selected institutions were abandoned, and four additional institutions were approached based on personal contacts. Two of these agreed to participate.

In two of the institutions who agreed to participate all students were invited to the survey, an in one institution only students from the area of science and technology were invited. For the remaining two institutions it is unknown if the institutions invited all or only some of the classes to the survey. For students below the age of 18 the institutions collected parental consent based on instructions from the Portuguese group (see also Appendix C).

Lithuania: Only students above the age of 18 were invited to participate in the survey. To comply with this restriction, only the students who were in their senior year were recruited. A cohort of institutions that were selected through the random draw was approached and directors and/or administrators of these institutions were asked to distribute the links to the questionnaire via email or e-diary to all the students older than 18 years old. To reach a sufficient number of participants, three cohorts of institutions were consecutively approached.

Slovenia: Only students above the age of 18 were included in the survey in Slovenia. To comply with this restriction, we only approached students in their senior year, and specifically asked them not to take the survey if they were below the mentioned age. Initially, ten schools chosen through a random draw was approached and given a choice to either send out an e-mail-invitation with a link to the online survey or to host a seminar where a researcher from the University of Ljubljana would present the INTEGRITY project and the topic of academic integrity, and the students would fill out the questionnaire in class. Two institutions accepted to participate, both of these chose to send out e-mail invitation to all of their senior year students.

The COVID-19 crisis made it very difficult to recruit further institutions in the in Autumn 2020. A total of five more institutions were recruited. Of these, three were from the random draw and two were recruited through personal contacts in order to get a better regional

distribution of the collected data. In three of these our personal contacts ran the questionnaire during their classes. The majority of the data were collected in this way. In two schools, the students were sent an e-mail invitation to all of their senior year students with the link to the survey, but relatively very few decided to participate in the study. In October 2020 we further distributed the invitation to the survey via our Facebook page.

Switzerland (FR): Only students above the age of 18 years were approached. Since the French-speaking part of Switzerland is a very small region we followed a total population sampling strategy to get a sufficient number of participants. Heads of the institutions were contacted. Institutions that agreed to participate would send out an e-mail invitation with a link to the survey to all their students above the age of 18 years. The students were asked to fill the questionnaire outside of school hours.

Ireland: Students of all ages, including below the age of 18 were recruited. A random draw of 51 institutions were made and these institutions were contacted in order and invited to participated in the survey. The target number of 200 participants was reached when the 39 first institutions had been invited, and no contact was made to the remaining 12 institutions on the list. Institutions who agreed to participate in the survey send out an e-mail invited to all their students. For students below the age of 18 a parental consent was collected and only students with parental consent were allowed to participate.
