# Supplementary material for: Lack of ethics or lack of knowledge? European upper secondary students’ doubts and misconceptions about integrity issues
Source: Int J Educ Integr. 2022 Aug 11;18(1):20. doi: 10.1007/s40979-022-00113-0 (PMC9365441; doi:10.1007/s40979-022-00113-0)
Supplement: Supplementary file 2 — Additional file 2. Descriptive statistics for the predictor variables [file 40979_2022_113_MOESM2_ESM.pdf]

Additional file 2: Descriptive statistics for the predictor variables

Table 1. Descriptive statistics for the predictor variables inserted in the mixed effects models

**Variables inserted as qualitative predictors (n=1654)**	proportions
**Age**	
below 18 years	0.27
18-19 years	0.50
>19 years	0.22
**Reported gender**	
Male	0.32
Female	0.58
None of the above/prefer not to answer	0.10
**Participated in one or more dedicated courses/lectures**	
no	0.70
yes	0.30
**Participated in one or more dedicated e-learning sessions**	
no	0.95
Yes	0.05
**Dedicated training by supervisors/teachers in other courses that commented on written work or assignment**	
No	0.56
Yes	0.44
**Dedicated training through courses not dedicated exclusively to such issues**	
no	0.80
yes	0.20
**Dedicated training through discussions with teachers outside regular classes**	
no	0.83
yes	0.17

**Variables inserted as continuous predictors (n)**	M	SD	MAX	MIN
Perception of questionable peer behavior (1450)*	-0.02	0.80	2.11	-2.39
Self-reported level of knowledge (1654)**.	-0.02	0.17	0.87	-2.52
Uncertainty about data collection and analysis (1171)***	1.03	1.04	3.00	0.00
Uncertainty about citation and plagiarism (1571)***	0.95	0.96	3.00	0.00
Uncertainty about collaboration and authorship (1460)***	0.83	0.98	3.00	0.00

*See Additional file 7 for details about this construct
** See Additional file 8 for details about this construct
***Single-item indicator. Question formulation was: "Over the past 12 months, have you been in a situation where you were unsure how to behave in an ethically correct manner in relation to". Response options were 0 = No, 1=Yes, once, 2=Yes, a few times, 3=Yes, many times"
