# Supplementary material for: Lack of ethics or lack of knowledge? European upper secondary students’ doubts and misconceptions about integrity issues
Source: Int J Educ Integr. 2022 Aug 11;18(1):20. doi: 10.1007/s40979-022-00113-0 (PMC9365441; doi:10.1007/s40979-022-00113-0)
Supplement: Supplementary file 3 — Additional file 3. Ethical approval [file 40979_2022_113_MOESM3_ESM.pdf]

Additional file 3: Ethical approval

The study was reviewed and approved by the IRB at the University of Copenhagen prior to the pilot tests. In the four other countries (Ireland, Portugal, Switzerland and Slovenia) local ERBs reviewed and approved recruitment plans for specific countries prior to data collection. In Lithuania research ethics committee (REC) review is only carried out for the studies falling within the scope of the Law on Ethics of Biomedical Research. This study was not within the scope of the mentioned law and therefore has not been eligible for the review and approval by RECs.

Participation in the study was voluntary and anonymous. Participants were not compensated for participating in the study.

Informed consent to participate was collected through the first question in the questionnaire (see Appendix E). The survey was terminated after this question for participants who did not give consent.

In Portugal and Ireland participants below the age of 18 were recruited. In both countries parental consent was collected and kept by the participating institutions following instructions send by members of the INTEGRITY team.

As the second question in the questionnaire, participants were asked about their age. Participants below the age of 18 were asked: “Has a parent or legal guardian agreed to let you participate in this survey?”. The survey was terminated for participants below the age of 18 who did not answer “yes” to this question.
