# Supplementary material for: Lack of ethics or lack of knowledge? European upper secondary students’ doubts and misconceptions about integrity issues
Source: Int J Educ Integr. 2022 Aug 11;18(1):20. doi: 10.1007/s40979-022-00113-0 (PMC9365441; doi:10.1007/s40979-022-00113-0)
Supplement: Supplementary file 4 — Additional file 4. Development, testing and translation of questionnaire [file 40979_2022_113_MOESM4_ESM.pdf]

Additional file 4: Development, testing and translation of questionnaire

The survey was conducted as part of the INTEGRITY project and framed on accordance with the general empirical aims of the project, stated in the project application:

1. **identify, map and evaluate** the existing understanding of students and early career researchers in terms of academic and research integrity and key concepts in relation to questionable research practice and research misconduct, including fabrication, falsification, and plagiarism via a new survey tool developed for this purpose;
2. **map and categorise** the grey area issues relating to good scientific practice that students and early career researchers are most likely to face in their daily practice, and the extent to which these vary across cultural and disciplinary backgrounds.

These general aims were translated into 15 different research questions. Based on these an explorative interview study of 72 students (18 upper secondary, 18 Bachelor's and 36 PhD) from Hungary, Denmark and Ireland was conducted (see Goddiksen et al. 2020 for details).

On basis of the interview study, the existing literature and the research questions a pilot survey was developed (in English) by the team at the University of Copenhagen and circulated to the partners in all participating countries. The partners commented on the survey and made sure that all questions were meaningful in the didactical context given in their country. The questionnaire was revised, set up online and pilot tested.

Two types of pilot tests were performed:

1. A small qualitative test involving a handful of Danish undergraduate and upper secondary school students. These participants took the survey while a member from the Copenhagen team was present. The participants were asked to comment on the questions as they answered the survey and debriefed on what they found difficult or unclear after the survey was finished.
2. A larger quantitative test. The pilot questionnaire was translated into Dutch, Lithuanian and Portuguese, and all versions were set up online. 550 participants recruited using convenience sampling completed the questionnaire.

Data from the pilot test was analysed for indications of questions being too difficult or too similar to other questions to allow for meaningful analysis. Based on this analysis and the general experience running the pilot test a number of questions were cut from the questionnaire, others were merged, and the language was generally simplified.

The final English version of the questionnaire was translated into the prevailing languages in the nine countries initially participating in the study (Denmark, Ireland, Lithuania, Portugal, Slovenia, Switzerland, Germany, the Netherlands, and Hungary) using a translation-back-translation approach: For each language, the English version of the questionnaire was first translated into the intended language, then back-translated into English by an independent translator without access to the original. A member of the Copenhagen team would then compare the back-translation with the original English version. Any discrepancies between the two versions of the questionnaire were then discussed with the relevant partners and the translated versions were adjusted where necessary.

When all translations were ready the online survey was set such that participants in any given country could choose between an English and a version translated into the dominant language of their country. Participants from the French speaking part of Switzerland were given the choice between French and English and participants from the German speaking part of Switzerland could choose between German and English. In each country native speaking partners thoroughly and systematically tested the local version of the survey for language and technical errors and all issues were addressed before the survey was send to participants.

See Goddiksen et. al (in review): Do European Undergraduate Students Understand the Grey Zones between Good Academic Practice and Misconduct? for further details on the development of the questionnaire.
