# Supplementary material for: Lack of ethics or lack of knowledge? European upper secondary students’ doubts and misconceptions about integrity issues
Source: Int J Educ Integr. 2022 Aug 11;18(1):20. doi: 10.1007/s40979-022-00113-0 (PMC9365441; doi:10.1007/s40979-022-00113-0)
Supplement: Supplementary file 5 — Additional file 5. English version of the questionnaire [file 40979_2022_113_MOESM5_ESM.pdf]

Additional file 5: English version of the questionnaire

Q#	Text	Answer options	Rules for the questions
**1.1**	[Language. This question is standard in the survey program. Only the answer options need translation.]	[English], [Local language], [Local language 2 (only if needed)]	If [Local language 1] run Local language 1 survey. If [Local language 2] run Local language 2 survey. If [English] run English survey
	This survey is about appropriate and inappropriate ways to behave as a researcher or student. Maybe you have heard about someone who behaved inappropriately, for instance by copying text from others, receiving unauthorised help, or by presenting inadequate or false results in order to make a conclusion look better. We are interested in understanding what you know and think about this because we want to improve teaching.<br>The survey will take about 20 minutes to complete.		
**1.2**	The present survey is completely anonymous. It will not be possible for anyone to trace your answers back to you. You can choose to leave the survey at any point before the survey is completed after which we will not use your responses. Once you have completed the survey you cannot withdraw your answers because they are anonymous.<br><br>About the survey<br>The survey was developed by an international team including researchers from XXX. It is part of a research project called INTEGRITY, which is funded by the EU through Horizon 2020. The results of the survey will be published in international research journals	[I understand the information provided to me and wish to participate in the survey], [I do not wish to participate in the survey]	If [I do not wish to participate in the survey] jump to PX
	Before we proceed to questions about appropriate and inappropriate practice, we would first like to ask some general questions about you		
**1.3**	What is your age?	Numerical response<br>[15-99]	
**1.3.1**	Has a parent or legal guardian agreed to let you participate in this survey?	[Yes], [No],<br>[I don’t know]	For participants under 18.<br>Activation rule: [<18] in Q1.2. If [No] or [I don’t know] jump to PX

Q#	Text	Answer options	Rules for the questions
**1.5**	In which country are you primarily studying?	[Denmark], [Germany], [Lithuania], [Hungary], [Ireland], [The Netherlands], [Portugal], [Slovenia], [Switzerland], [Other]	If [Other] jump to PX
**1.6**	What is your current level of study?	*The specific formulation is left to the translator*. General definitions: [High school] Student in a program that is a sufficient requirement for entering one or more university educations. The program cannot itself be a university education. [BA] Student in the first cycle (see http://www.ehea.info/page-three-cycle-system) defined in the Bologna declaration, enrolled in an institution that also offers Ph.D. level education, having earned at least 60 and less than 180 ECTS points. [Master's student] Student in the second cycle defined in the Bologna declaration, having earned more than 180 ECTS points. [Research master] Student in the second cycle defined in the Bologna declaration, having earned more than 180 ECTS points, enrolled in a program that involves work on a research project that will typically be continued in a Ph.D.-project. [Ph.D. student] Student in the third cycle defined in	If [Master student] or [Other] jump to PX

Q#	Text	Answer options	Rules for the questions
		the Bologna declaration. Students at this level are preparing a dissertation based on one or more research projects guided by one or more senior researchers, [Other]	
**1.6.3a**	At which of the following institutions are you enrolled?	[List generated from data collection plan] + [None of the above]	Jump to Px if [None of the above]
**1.8a**	As part of your education, do you sometimes:<br><br>[1.8a.1] Work with other students to prepare written work, oral presentations or to perform laboratory work?<br><br>[1.8a.2] Collect information through either laboratory work, surveys, interviews or field work?	[Yes], [No]	
**2.0**	In the next part of the survey, we will focus on your experience and knowledge of how to behave in some of the difficult situations that students and Ph.D. students might encounter. You will be asked about appropriate behaviour in relation to drawing on other people's work, when working with others and when collecting, analysing and presenting data.		The text was given in different versions depending on the answer to 1.8a
**2.1**	To what extent do you agree with the following claim:<br>I have a good understanding of the official standards of good practice that apply to me in relation to...<br><br>Examples of “official standards of good practice” could be rules and regulations stating what is prohibited, but it could also include guidelines and codes of conduct describing how to behave correctly.<br><br>[2.1.1] ... citation and plagiarism<br>[2.1.2] … working with others and assigning authorship<br>[2.1.3] ... collection, analysis and presentation of data	[Fully agree], [Agree], [Neutral], [Disagree], [Fully disagree], [I don’t know]	All students were asked [2.1.1].<br>Only students who answered [Yes] to [1.8a.1] were given [2.1.2].<br>Only students who answered [Yes] to [1.8a.2] were given [2.1.3].
**2.2**	To what extent do you agree with the following claim:<br>In general, I know how to behave in an ethically correct manner in relation to ...	[Fully agree], [Agree], [Neutral], [Disagree], [Fully disagree], [I don’t know]	All students were asked [2.2.1].<br>Only students who answered [Yes] to [1.8a.1] were given [2.2.2].

Q#	Text	Answer options	Rules for the questions
	[2.2.1] ... citation and plagiarism<br>[2.2.2] … working with others and assigning authorship<br>[2.2.3] ... collection, analysis and presentation of data		Only students who answered [Yes] to [1.8a.2] were given [2.2.3].
**2.3**	Over the past 12 months, have you been in a situation where you were unsure how to behave in an ethically correct manner in relation to…<br><br>[2.3.1] ... citation and plagiarism<br>[2.3.2] … working with others and assigning authorship<br>[2.3.3] ... collection, analysis and presentation of data	[Yes, many times], [Yes, a few times], [Yes, once], [No], [Not applicable]	All students were asked [2.1.1].<br>Only students who answered [Yes] to [1.8a.1] were given [2.3.2].<br>Only students who answered [Yes] to [1.8a.2] were given [2.3.3].
**2.4ab**	A friend of yours wants to use a paragraph from a textbook in an assignment they are currently writing. The paragraph is from page 10 in a book written by J. Brown in 1981. In the book, the paragraph reads:<br><br>"Process Y was first discovered in 1931 by the German scientist H. Neumann, and was refined in the late 50s. Its full importance was, however, only fully understood when it was described in the seminal paper by D. Dirksen in 1971."<br><br>Your friend can incorporate the paragraph into the introduction of their own text in various ways. Some of these are presented on the next pages. For each version, please indicate whether or not you believe your friend has acted in a way that is acceptable.<br><br>[Q2.4ab.1] First version: In this assignment, I will focus on a new application of process Y. As is well known, the process was first discovered in 1931 by the German scientist H. Neumann, and was refined in the late 50s. Its full importance was, however, only fully understood when it was described in the seminal paper by D. Dirksen in 1971, and the process' commercial importance was discovered. In this paper, I will continue this work by showing how process Y can be used in the production of…<br><br>The original by Brown:<br>"Process Y was first discovered in 1931 by the German scientist H. Neumann, and was refined in the late 50s. Its full importance was, however, only fully understood when it was described in the seminal paper by D. Dirksen in 1971."	[Completely acceptable], [Acceptable], [Neutral], [Unacceptable], [Completely unacceptable], [I don't know]	

Q#	Text	Answer options	Rules for the questions
	Please indicate whether or not you believe your friend has acted in a way that is acceptable.<br><br>[Q2.4ab.2] Second version: In this assignment, I will focus on a new application of process Y. As is well known, the process was first identified in 1931 by the German scientist H. Neumann, and was later refined. Its full usefulness was, however, only fully understood when it was described in the important paper by D. Dirksen in 1971, and the process' commercial importance was discovered. In this paper, I will continue this work by showing how process Y can be used in the production of…<br><br>[Q2.4ab.3] Third version: In this assignment, I will focus on a new application of process Y. As is well known, the process was first identified in 1931 by the German scientist H. Neumann, and was later refined. Its full usefulness was, however, only fully understood when it was described in the important paper by D. Dirksen in 1971 (Brown, 1981), and the process' commercial importance was discovered. In this paper, I will continue this work by showing how process Y can be used in the production of…<br><br>[Q2.4ab.4] Fourth version: In this assignment, I will focus on a new application of process Y. According to Brown (1981:10), the process was first discovered in 1931, although its usefulness and commercial importance were only understood in the early 1970s. In this paper, I will continue this work by showing how process Y can be used in the production of…		
**2.6**	Please indicate whether you believe the following actions go against the official rules and regulations that apply to you in relation to plagiarism.<br>• [Q2.6.1] Copying an entire page stating a central point from an external source into your own text without quotation marks but including a reference.<br>• [Q2.6.2] Copying one short paragraph stating a central point from an external source into your own text without quotation marks but including a reference.<br>• [Q2.6.3] Changing 10% of the words in a short paragraph stating a central point from an external source and using it in your own text with a reference.	[Yes, it is a serious violation], [Yes, but it is not a serious violation], [No, it is not against the rules], [The rules are unclear], [It depends on the situation], [I don't know]	

Q#	Text	Answer options	Rules for the questions
	• [Q2.6.4] Copying a central point formulated in half a sentence from an external source without marking it with quotation marks but including a reference.		
**2.11ab**	Please indicate whether you believe the following actions are against the official rules and regulations that apply to you in relation to working with others and assigning authorship.<br>• [Q2.11ab.1] Paying someone to write an assignment for you.<br>• [Q2.11ab.2] Comparing answers to an individual assignment with other students before handing in the assignment.<br>• [Q2.11ab.3] Handing in an assignment that you made with extensive help from another student or family member without mentioning the help you received.<br>• [Q2.11ab.4] Let one member of a group do all the writing on a group project while the other members contribute to analysis and literature search.	[Yes, it is a serious violation], [Yes, but it is not a serious violation], [No, it is not against the rules], [The rules are unclear], [It depends on the situation], [I don't know]	
**2.17**	Please indicate whether you believe the following actions go against the rules and regulations that apply to you in relation to data collection, analysis and presentation.<br>• [Q2.17.1] Not mentioning in an assignment that you removed a number of deviating data points from a data set when the cause of the deviation was unknown.<br>• [Q2.17.2] Not mentioning in an assignment that you removed a number of deviating data points from a data set when the cause of the deviation was known.<br>• [Q2.17.3] Not mentioning in an assignment that you replaced a number of outliers in a data set with data points obtained through estimates based on the remaining data points.	[Yes, it is a serious violation], [Yes, but it is not a serious violation], [No, it is not against the rules], [The rules are unclear], [It depends on the situation], [I don't know]	
	In the following section, we will ask you some questions about how and where you obtained your knowledge about good practice.		
**3.1**	Have you taken courses on rules and/or ethically correct behaviour in relation to the themes introduced above during your current or previous studies? (Multiple answers possible)	[Yes, one or more dedicated courses], [Yes, one or more lectures], [Yes, one or more dedicated e-learning sessions], [No]	
**3.1.1**	Approximately how many working days have you spent on such courses in total?	[Less than 1 working day], [1 full working day], [1-3 full working days], [3-5 full working days], [1-2 working	For participants who answered [Yes, one or more dedicated courses], [Yes, one or more lectures], or [Yes, one or

Q#	Text	Answer options	Rules for the questions
		weeks], [More than 2 working weeks], [I do not recall]	more dedicated e-learning sessions] in Q3.1
**3.2a**	Have you learned about rules and/or ethically correct behaviour in relation to the themes introduced above through any other method?<br>(Multiple answers possible)	[Yes, through supervisors/teachers in other courses that commented on my written work or assignments], [Yes, through courses not dedicated exclusively to such issues], [Yes, through discussions with fellow students], [Yes, through discussions with teachers outside regular classes], [Yes, through self-study], [Yes, through discussions with friends and family outside my institution], [Yes, other], [No], [I don't know]	
**3.3a**	Below are a number of topics related to appropriate and inappropriate ways of behaving as a student. Please indicate the **three topics** you think are the most important when such themes are taught to your classmates	[Drawing on other peoples' work (including plagiarism)], [Working with others], [Collecting, analysing and presenting data],<br>[Pressure from teachers or others in power], [Conflicts of loyalty], [How to deal with violations of rules and allegations of cheating], [Learning culture (e.g. elements in the learning environment that promote or hinder ethical behavior)], [Other topics], [I don't think teaching about such themes is relevant], [I don't know]	
	It is one thing to know the right thing to do in a given situation, but another to actually do it. You have probably heard rumours about researchers and/or students who		

Q#	Text	Answer options	Rules for the questions
	plagiarised, manipulated data or deviated from ideal practice in other ways. We are interested in knowing how common you think such deviations are among your peers and whether you yourself have deviated from best practice. We remind you that this survey is entirely anonymous; no one will be able to identify your specific answers to these questions.		
**4.1a**	To what extent do you agree with the following statements?<br>It is common for my classmates to...<br>• [Q4.1a.1] ... delete data from an experiment only because it somehow seemed wrong.<br>• [Q4.1a.2] ... give a misleading or dubious interpretation of texts, works of art or interview data in order to achieve results the teacher will accept.<br>• [Q4.1a.3] … receive help from other students or family members on assignments they were supposed to complete on their own.<br>• [Q4.1a.4] ... copy shorter passages from other sources into their own texts without marking them as quotes.<br>• [Q4.1a.5] ... add students as co-authors of group assignments, even though they did not contribute.	[Fully agree], [Agree], [Neutral], [Disagree], [Fully disagree], [I don't know]	
**4.2a**	During your high-school education, have you...<br>(We remind you that this survey is entirely anonymous; no one will be able to identify your specific answers to these questions)<br>• [Q4.2a.1] ... deleted deviating data points based on a gut feeling that they were inaccurate.<br>• [Q4.2a.2] … received help from other students or family members on assignments you were supposed to complete on your own<br>• [Q4.2a.3] ... copied shorter passages from other sources into your own text without marking them as quotes.<br>• [Q4.2a.4] … added students as co-authors of group assignments, even though they did not contribute.<br>• [Q4.2a.5] … refused to help a friend with his or her assignment because you were unsure whether it was allowed.<br>• [Q4.2a.6] … worried about being accused of plagiarism based on an automatic plagiarism check, even though you did not intentionally plagiarise.	[Yes, many times], [Yes, a few times], [Yes, once], [No], [I prefer not to answer], [Not applicable], [I don't know]	

Q#	Text	Answer options	Rules for the questions
	The last questions are about *why* people deviate from best practice. There may of course be many reasons, but we are interested in knowing which ones you think are the most important.		
**5.1ab**	To what extent do you agree with the following statements?<br>If students at my institution deviate from what is ethically acceptable (for instance by plagiarising or putting their name on work they did not contribute to), they do it because...<br>• [Q5.1ab.1] ... they are lazy.<br>• [Q5.1ab.2] ... their teacher or other people in power tell them to.<br>• [Q5.1ab.3] ... they fear they will not be awarded their degree if they don't.<br>• [Q5.1ab.4] ... they think it is very unlikely that they will get caught.<br>• [Q5.1ab.5] ... they think everyone else does it.<br>• [Q5.1ab.6] ... their friends and/or family encourage it directly or indirectly.<br>• [Q5.1ab.7] ... they want to be the best.<br>• [Q5.1ab.8] ... they have not been properly informed what the ethically correct behaviour is.<br>• [Q5.1ab.9] … they fear that they will not be able to pursue a specific education if they don't.<br>• [Q5.1ab.10] … other reasons.	[Fully agree], [Agree], [Neutral], [Disagree], [Fully disagree], [I don't know], [Not applicable]	
**6.0 [1.4]**	Before you submit your data we would like to ask you one final question:<br>Which gender do you primarily identify with?	[Male], [Female], [None of the above], [I prefer not to answer]	
	The survey is now finished. Thank you very much for your participation!<br>If you are interested in the results of this survey, you will be able to find them at http://h2020integrity.eu/ as soon as they are available.		
**PX**	Unfortunately, you are outside the target group of this survey. Thank you for your time. If you are interested in the results of this survey, you will be able to find them at http://h2020integrity.eu/ as soon as they are available.		
