# Supplementary material for: Lack of ethics or lack of knowledge? European upper secondary students’ doubts and misconceptions about integrity issues
Source: Int J Educ Integr. 2022 Aug 11;18(1):20. doi: 10.1007/s40979-022-00113-0 (PMC9365441; doi:10.1007/s40979-022-00113-0)
Supplement: Supplementary file 6 — Additional file 6. Data stratified by country [file 40979_2022_113_MOESM6_ESM.pdf]

Additional file 6: Data stratified by country

Table 1: "I have a good understanding of the official standards of good practice that apply to me in relation to citation and plagiarism" (n=1654)

	Fully agree	Agree	Neutral	Disagree	Fully disagree	I don't know
Denmark	26.6%	46.6%	18.8%	4.7%	1.3%	2.1%
Ireland	17.9%	32.4%	20.3%	9.3%	3.1%	16.9%
Lithuania	22.6%	41.1%	22.0%	5.4%	6.0%	3.0%
Portugal	17.9%	30.3%	21.6%	16.5%	11.5%	2.3%
Switzerland (FR)	31.3%	43.4%	13.0%	6.5%	4.5%	1.4%
Slovenia	14.6%	53.6%	13.8%	7.1%	3.8%	7.1%

Table 2: "I have a good understanding of the official standards of good practice that apply to me in relation to working with others and assigning authorship" (n=1551)

	Fully agree	Agree	Neutral	Disagree	Fully disagree	I don't know
Denmark	15.9%	47.9%	26.6%	4.1%	1.1%	4.4%
Ireland	27.8%	46.9%	15.2%	3.2%	0.0%	6.9%
Lithuania	24.1%	53.2%	17.7%	1.9%	1.3%	1.9%
Portugal	24.9%	50.7%	14.6%	3.4%	5.4%	1.0%
Switzerland (FR)	33.3%	37.4%	16.2%	7.0%	2.3%	3.8%
Slovenia	14.9%	51.2%	18.9%	10.0%	2.5%	2.5%

Table 3: "I have a good understanding of the official standards of good practice that apply to me in relation to collection. analysis and presentation of data" (n=1220)

	Fully agree	Agree	Neutral	Disagree	Fully disagree	I don't know
Denmark	18.0%	63.7%	14.1%	2.5%	0.0%	1.8%
Ireland	24.9%	49.8%	17.5%	1.9%	0.8%	5.1%
Lithuania	31.3%	52.8%	11.8%	2.1%	0.0%	2.1%
Portugal	33.5%	55.9%	8.2%	0.0%	1.8%	0.6%
Switzerland (FR)	32.0%	42.5%	15.1%	6.4%	0.0%	4.1%
Slovenia	19.2%	61.6%	13.0%	3.4%	0.7%	2.1%

Table 4: "In general, I know how to behave in an ethically correct manner in relation to citation and plagiarism" (n=1654)

	Fully agree	Agree	Neutral	Disagree	Fully disagree	I don't know
Denmark	33.1%	47.1%	14.8%	3.9%	0.3%	0.8%
Ireland	20.0%	38.6%	19.0%	5.2%	3.4%	13.8%
Lithuania	25.0%	44.0%	20.8%	3.6%	2.4%	4.2%
Portugal	18.3%	32.6%	23.4%	17.0%	7.3%	1.4%
Switzerland (FR)	33.2%	43.9%	12.1%	5.9%	3.4%	1.4%
Slovenia	15.1%	56.9%	12.1%	6.7%	2.9%	6.3%

Table 5: "In general, I know how to behave in an ethically correct manner in relation to working with others and assigning authorship" (n=1551)

	Fully agree	Agree	Neutral	Disagree	Fully disagree	I don't know
Denmark	25.8%	49.6%	17.5%	4.4%	0.3%	2.5%
Ireland	32.5%	43.0%	17.7%	2.2%	0.7%	4.0%
Lithuania	29.1%	50.0%	12.7%	5.7%	0.0%	2.5%
Portugal	28.3%	47.3%	14.6%	5.4%	3.4%	1.0%
Switzerland (FR)	33.9%	38.6%	15.9%	6.4%	2.0%	3.2%
Slovenia	15.9%	54.7%	16.4%	7.0%	2.0%	4.0%

Table 6: "In general, I know how to behave in an ethically correct manner in relation to collection, analysis and presentation of data" (n=1220)

	Fully agree	Agree	Neutral	Disagree	Fully disagree	I don't know
Denmark	26.1%	54.9%	16.2%	1.8%	0.0%	1.1%
Ireland	28.0%	46.7%	19.5%	2.3%	0.4%	3.1%
Lithuania	28.5%	51.4%	14.6%	3.5%	0.0%	2.1%
Portugal	32.4%	51.2%	11.8%	1.8%	2.4%	0.6%
Switzerland (FR)	32.0%	42.9%	14.6%	6.8%	0.5%	3.2%
Slovenia	17.1%	63.7%	14.4%	2.1%	0.7%	2.1%

Table 7: "Over the past 12 months, have you been in a situation where you were unsure how to behave in an ethically correct manner in relation to citation and plagiarism" (n=1654)

	Yes, many times	Yes, a few times	Yes, once	No	Not applicable
Denmark	3.4%	32.3%	22.9%	38.8%	2.6%
Ireland	7.2%	22.4%	17.2%	45.9%	7.2%
Lithuania	3.0%	23.8%	17.3%	49.4%	6.5%
Portugal	4.1%	18.8%	13.3%	53.7%	10.1%
Switzerland (FR)	7.3%	31.0%	27.0%	31.0%	3.7%
Slovenia	4.6%	23.8%	29.7%	39.3%	2.5%

Table 8: "Over the past 12 months, have you been in a situation where you were unsure how to behave in an ethically correct manner in relation to working with others and assigning authorship" (n=1654)

	Yes, many times	Yes, a few times	Yes, once	No	Not applicable
Denmark	4.9%	23.8%	18.4%	48.2%	4.7%
Ireland	7.6%	30.7%	20.2%	37.9%	3.6%
Lithuania	2.5%	24.7%	19.0%	48.7%	5.1%
Portugal	5.9%	31.7%	11.2%	44.4%	6.8%
Switzerland (FR)	2.9%	19.1%	12.8%	55.9%	9.3%

Slovenia	4.0%	14.4%	13.4%	63.2%	5.0%

Table 9: "Over the past 12 months, have you been in a situation where you were unsure how to behave in an ethically correct manner in relation to collection, analysis and presentation of data" (n=1654)

	Yes, many times	Yes, a few times	Yes, once	No	Not applicable
Denmark	5.3%	20.8%	22.9%	47.5%	3.5%
Ireland	11.3%	31.1%	17.9%	37.4%	2.3%
Lithuania	8.3%	29.2%	11.1%	48.6%	2.8%
Portugal	12.9%	35.9%	12.4%	32.4%	6.5%
Switzerland (FR)	10.0%	25.1%	23.3%	35.2%	6.4%
Slovenia	4.1%	23.3%	19.9%	50.0%	2.7%

Table 10: Friends use of a paraphrase from a textbook: First paraphrase. "Please indicate whether or not you believe your friend has acted in a way that is acceptable" (n=1654)

	Completely acceptable	Acceptable	Neutral	Unaccept-able	Completely unaccept-able	I don't know
Denmark	13.3%	42.2%	19.3%	14.3%	2.6%	8.3%
Ireland	10.0%	30.0%	29.0%	15.2%	3.8%	12.1%
Lithuania	10.7%	36.9%	26.8%	15.5%	3.0%	7.1%
Portugal	13.3%	33.9%	17.9%	25.7%	5.5%	3.7%
Switzerland (FR)	7.3%	15.2%	16.1%	35.2%	20.6%	5.6%
Slovenia	8.4%	33.9%	18.8%	24.3%	5.9%	8.8%

Table 11: Friends use of a paraphrase from a textbook: Second paraphrase. “Please indicate whether or not you believe your friend has acted in a way that is acceptable” (n=1654)

	Completely acceptable	Acceptable	Neutral	Unaccept-able	Completely unaccept-able	I don’t know
Denmark	13.0%	39.6%	24.0%	10.7%	1.6%	11.2%
Ireland	10.7%	34.8%	31.7%	7.9%	0.3%	14.5%
Lithuania	9.5%	35.1%	32.7%	15.5%	0.6%	6.5%
Portugal	9.6%	37.6%	28.0%	17.4%	2.3%	5.0%
Switzerland (FR)	6.2%	22.3%	26.8%	31.5%	6.2%	7.0%
Slovenia	6.7%	35.1%	28.9%	17.2%	2.1%	10.0%

Table 12: Friends use of a paraphrase from a textbook: Third paraphrase. “Please indicate whether or not you believe your friend has acted in a way that is acceptable” (n=1654)

	Completely acceptable	Acceptable	Neutral	Unaccept-able	Completely unaccept-able	I don’t know
Denmark	21.6%	39.3%	18.2%	6.3%	2.3%	12.2%
Ireland	12.4%	30.7%	30.7%	7.9%	0.7%	17.6%
Lithuania	13.7%	33.9%	33.3%	7.7%	3.0%	8.3%
Portugal	20.2%	35.8%	28.9%	9.6%	1.4%	4.1%
Switzerland (FR)	14.9%	29.9%	25.6%	16.6%	4.2%	8.7%
Slovenia	16.7%	39.7%	20.9%	9.2%	1.3%	12.1%

Table 13: Friends use of a paraphrase from a textbook: Fourth paraphrase. “Please indicate whether or not you believe your friend has acted in a way that is acceptable” (n=1654)

	Completely acceptable	Acceptable	Neutral	Unaccept-able	Completely unaccept-able	I don’t know
Denmark	19.8%	31.5%	22.1%	8.6%	1.8%	16.1%
Ireland	20.7%	24.8%	24.5%	9.0%	1.4%	19.7%
Lithuania	13.1%	26.2%	25.6%	16.1%	4.2%	14.9%
Portugal	27.1%	30.7%	23.9%	8.7%	4.1%	5.5%
Switzerland (FR)	27.0%	33.5%	20.0%	9.6%	2.0%	7.9%
Slovenia	26.8%	37.7%	19.7%	6.3%	1.3%	8.4%

Table 14: “Please indicate whether you believe the following actions go against the official rules and regulations that apply to you in relation to plagiarism: Copying an entire page stating a central point from an external source into your own text without quotation marks but including a reference” (n=1654)

	Yes, it is a serious violation	Yes, but it is not a serious violation	No, it is not against the rules	The rules are unclear	It depends on the situation	I don’t know
Denmark	69.0%	17.2%	5.5%	1.8%	3.6%	2.9%
Ireland	34.1%	29.3%	11.4%	3.8%	10.7%	10.7%
Lithuania	26.2%	29.8%	16.1%	4.2%	15.5%	8.3%
Portugal	48.2%	29.4%	2.8%	3.7%	11.0%	5.0%

Switzerland (FR)	55.5%	29.6%	4.5%	3.1%	4.8%	2.5%
Slovenia	36.0%	35.1%	11.7%	5.9%	5.4%	5.9%

Table 15: "Please indicate whether you believe the following actions go against the official rules and regulations that apply to you in relation to plagiarism: Copying one short paragraph stating a central point from an external source into your own text without quotation marks but including a reference" (n=1654)

	Yes, it is a serious violation	Yes, but it is not a serious violation	No, it is not against the rules	The rules are unclear	It depends on the situation	I don't know
Denmark	21.9%	47.1%	18.0%	2.9%	5.7%	4.4%
Ireland	7.9%	40.0%	25.5%	5.2%	11.4%	10.0%
Lithuania	11.3%	36.9%	28.6%	2.4%	8.9%	11.9%
Portugal	11.5%	46.8%	18.3%	6.0%	11.9%	5.5%
Switzerland (FR)	27.0%	48.2%	11.3%	2.3%	7.0%	4.2%
Slovenia	12.1%	44.4%	23.4%	5.4%	6.7%	7.9%

Table 16: "Please indicate whether you believe the following actions go against the official rules and regulations that apply to you in relation to plagiarism: Changing 10% of the words in a short paragraph stating a central point from an external source and using it in your own text with a reference" (n=1654)

	Yes, it is a serious violation	Yes, but it is not a serious violation	No, it is not against the rules	The rules are unclear	It depends on the situation	I don't know
Denmark	13.0%	26.3%	32.3%	9.4%	8.6%	10.4%
Ireland	8.6%	23.8%	35.5%	4.8%	14.1%	13.1%
Lithuania	18.5%	22.6%	28.6%	6.0%	8.9%	15.5%
Portugal	7.3%	28.4%	36.7%	6.4%	13.3%	7.8%
Switzerland (FR)	13.0%	36.6%	31.3%	6.8%	7.3%	5.1%
Slovenia	7.1%	21.3%	41.4%	10.0%	7.5%	12.6%

Table 17: "Please indicate whether you believe the following actions go against the official rules and regulations that apply to you in relation to plagiarism: Copying a central point formulated in half a sentence from an external source without marking it with quotation marks but including a reference" (n=1654)

	Yes, it is a serious violation	Yes, but it is not a serious violation	No, it is not against the rules	The rules are unclear	It depends on the situation	I don't know
Denmark	10.9%	20.3%	31.3%	9.9%	10.4%	17.2%
Ireland	8.6%	15.9%	32.4%	8.6%	12.1%	22.4%
Lithuania	13.1%	22.6%	31.0%	4.8%	10.1%	18.5%
Portugal	6.9%	22.0%	34.4%	7.8%	14.7%	14.2%
Switzerland (FR)	9.0%	24.2%	40.3%	8.2%	8.7%	9.6%
Slovenia	9.2%	26.4%	32.6%	8.4%	9.6%	13.8%

Table 18: "Please indicate whether you believe the following actions go against the official rules and regulations that apply to you in relation to working with others and assigning authorship: Paying someone to write an assignment for you" (n=1654)

	Yes, it is a serious violation	Yes, but it is not a serious violation	No, it is not against the rules	The rules are unclear	It depends on the situation	I don't know
Denmark	84.1%	5.7%	2.9%	1.6%	1.8%	3.9%
Ireland	73.4%	7.6%	6.2%	3.1%	4.5%	5.2%
Lithuania	55.4%	14.9%	11.9%	2.4%	5.4%	10.1%
Portugal	61.9%	9.6%	11.5%	2.8%	9.2%	5.0%
Switzerland (FR)	63.4%	12.7%	8.7%	7.9%	3.7%	3.7%
Slovenia	74.1%	10.9%	3.3%	1.7%	4.2%	5.9%

Table 19: "Please indicate whether you believe the following actions go against the official rules and regulations that apply to you in relation to working with others and assigning authorship: Comparing answers to an individual assignment with other students before handing in the assignment" (=1654)

	Yes, it is a serious violation	Yes, but it is not a serious violation	No, it is not against the rules	The rules are unclear	It depends on the situation	I don't know
Denmark	4.4%	12.5%	60.2%	7.3%	10.9%	4.7%
Ireland	17.6%	23.1%	34.1%	6.2%	13.1%	5.9%
Lithuania	11.3%	25.6%	35.1%	3.0%	13.7%	11.3%
Portugal	7.8%	16.5%	50.5%	6.9%	13.3%	5.0%
Switzerland (FR)	2.3%	13.8%	64.8%	6.8%	8.5%	3.9%
Slovenia	4.2%	13.8%	54.0%	8.8%	12.1%	7.1%

Table 20: "Please indicate whether you believe the following actions go against the official rules and regulations that apply to you in relation to working with others and assigning authorship: Handing in an assignment that you made with extensive help from another student or family member without mentioning the help you received" (n=1654)

	Yes, it is a serious violation	Yes, but it is not a serious violation	No, it is not against the rules	The rules are unclear	It depends on the situation	I don't know
Denmark	8.1%	22.4%	38.8%	10.4%	12.0%	8.3%
Ireland	17.9%	32.4%	21.4%	9.0%	11.7%	7.6%
Lithuania	18.5%	37.5%	17.9%	4.2%	12.5%	9.5%
Portugal	19.3%	28.0%	25.2%	9.6%	13.8%	4.1%
Switzerland (FR)	11.0%	23.9%	38.3%	12.1%	9.9%	4.8%
Slovenia	28.5%	33.1%	12.6%	7.9%	10.9%	7.1%

Table 21: "Please indicate whether you believe the following actions go against the official rules and regulations that apply to you in relation to working with others and assigning authorship: Let one member of a group do all the writing on a group project while the other members contribute to analysis and literature search" (n=1654)

	Yes, it is a serious violation	Yes, but it is not a serious violation	No, it is not against the rules	The rules are unclear	It depends on the situation	I don't know
Denmark	16.7%	24.5%	26.6%	11.2%	14.6%	6.5%
Ireland	16.2%	23.8%	33.8%	4.8%	14.1%	7.2%
Lithuania	9.5%	10.7%	48.8%	4.2%	18.5%	8.3%
Portugal	19.7%	18.8%	27.1%	7.8%	21.1%	5.5%
Switzerland (FR)	4.8%	11.3%	53.0%	9.0%	17.5%	4.5%
Slovenia	11.3%	15.9%	38.5%	10.0%	19.7%	4.6%

Table 22: "Please indicate whether you believe the following actions go against the official rules and regulations that apply to you in relation to data collection, analysis and presentation: Not mentioning in an assignment that you removed a number of deviating data points from a data set when the cause of the deviation was unknown" (n=1654)

	Yes, it is a serious violation	Yes, but it is not a serious violation	No, it is not against the rules	The rules are unclear	It depends on the situation	I don't know
Denmark	19.3%	23.4%	15.1%	8.9%	8.9%	24.5%
Ireland	20.7%	22.1%	10.0%	7.6%	4.5%	35.2%
Lithuania	14.3%	22.6%	19.6%	5.4%	11.9%	26.2%
Portugal	19.3%	16.1%	20.2%	10.1%	11.9%	22.5%
Switzerland (FR)	21.7%	26.2%	13.0%	11.5%	5.9%	21.7%
Slovenia	19.7%	33.5%	11.7%	7.9%	10.0%	17.2%

Table 23: "Please indicate whether you believe the following actions go against the official rules and regulations that apply to you in relation to data collection, analysis and presentation: Not mentioning in an assignment that you removed a number of deviating data points from a data set when the cause of the deviation was known" (n=1654)

	Yes, it is a serious violation	Yes, but it is not a serious violation	No, it is not against the rules	The rules are unclear	It depends on the situation	I don't know
Denmark	17.7%	32.8%	11.5%	7.6%	8.3%	22.1%
Ireland	20.7%	23.4%	9.3%	6.9%	6.6%	33.1%
Lithuania	16.7%	25.6%	18.5%	3.6%	11.9%	23.8%
Portugal	19.7%	17.0%	21.1%	8.7%	11.9%	21.6%
Switzerland (FR)	23.7%	20.3%	15.2%	11.3%	7.0%	22.5%
Slovenia	23.0%	27.2%	17.2%	4.6%	10.5%	17.6%

Table 24: "Please indicate whether you believe the following actions go against the official rules and regulations that apply to you in relation to data collection, analysis and presentation: Not mentioning in an assignment that you replaced a number of outliers in a data set with data points obtained through estimates based on the remaining data points" (n=1654)

	Yes, it is a serious violation	Yes, but it is not a serious violation	No, it is not against the rules	The rules are unclear	It depends on the situation	I don't know

Denmark	25.5%	24.7%	8.9%	8.6%	5.5%	26,8%
Ireland	20.7%	22.8%	10.7%	5.5%	5.2%	35,2%
Lithuania	15.5%	22.0%	14.3%	9.5%	7.1%	31,5%
Portugal	22.5%	23.9%	12.4%	7.8%	12.8%	20.6%
Switzerland (FR)	33.0%	19.7%	8.2%	8.7%	5.1%	25.4%
Slovenia	33.1%	28.5%	5.0%	6.3%	5.4%	21.8%

Table 25: Level of academic integrity training in dedicated and non-dedicated settings (n=1654)

	"Have you taken courses on rules and/or ethically correct behaviour in relation to the themes introduced above during your current or previous studies?"			"Have you learned about rules and/or ethically correct behaviour in relation to the themes introduced above through any other method?"		
	Yes, one or more lectures or courses	Yes, one or more dedicated e-sessions	No	Yes, through supervisors/ teachers in other courses that commented on my written work or assignment	Yes, through courses not dedicated exclusively to such issues	Yes, through discussions with teachers outside regular classes
Denmark	52.1%	6.3%	43.0%	44.0%	7.3%	20.6%
Ireland	16.9%	7.2%	76.2%	26.9%	25.9%	8.3%
Lithuania	20.8%	6.5%	75.0%	37.5%	19.6%	13.1%
Portugal	27.1%	4.1%	69.7%	44.0%	12.4%	16.5%
Switzerland (FR)	34.6%	3.7%	62.5%	48.7%	28.2%	19.4%
Slovenia	11.7%	2.5%	86.6%	59.4%	28.9%	20.1%

Table 26: Perceived questionable practices in peers: "It is common for my classmates to..."
(Shares of participants who answered "Fully agree" or "Agree") (n=1654)

	...delete data from an experiment only because it somehow seemed wrong	... copy shorter passages from other sources into their own texts without marking them as quotes	... add students as co-authors of group assignments, even though they did not contribute	... receive help from other students or family members on assignments they were supposed to complete on their own
Denmark	20.6%	31.3%	46.9%	58.9%
Ireland	30.0%	51.0%	36.9%	52.8%
Lithuania	26.2%	53.6%	48.2%	61.3%
Portugal	30.7%	45.0%	38.1%	56.9%
Switzerland (FR)	34.1%	41.4%	36.9%	73.8%
Slovenia	27.6%	49.0%	38.1%	58.6%

Table 27: Participants own engagement in questionable practices: "During your high-school education, have you..."
(Shares of participants who answered "Yes, many times", "Yes, a few times" or "Yes, once").

	…deleted data from an experiment only because it somehow seemed wrong (n=1358)	… copied shorter passages from other sources into your own text without marking them as quotes (n=1442)	… added students as co-authors of group assignments, even though they did not contribute (n=1442)	… received help from other students or family members on assignments you were supposed to complete on your own (n=1487)
Denmark	38.1%	49.6%	76.5%	80.2%
Ireland	54.0%	75.8%	55.6%	80.2%
Lithuania	58.6%	71.5%	70.3%	78.5%
Portugal	54.2%	72.0%	64.9%	82.9%
Switzerland (FR)	52.8%	45.0%	47.0%	77.7%
Slovenia	45.4%	63.2%	47.1%	84.2%

(Respondents who answered: “Not applicable”, “I prefer not to answer”, and “I don’t know” are not included in the statistics)
