# Supplementary material for: Lack of ethics or lack of knowledge? European upper secondary students’ doubts and misconceptions about integrity issues
Source: Int J Educ Integr. 2022 Aug 11;18(1):20. doi: 10.1007/s40979-022-00113-0 (PMC9365441; doi:10.1007/s40979-022-00113-0)
Supplement: Supplementary file 7 — Additional file 7. Assessment of measurement invariance of peer perception [file 40979_2022_113_MOESM7_ESM.pdf]

Additional file 7: Assessment of measurement invariance of peer perception

Table 1 shows the four questions that were used as input variables (manifest variables) in the confirmatory factor analysis (CFA). Response options ranged from 1 (fully agree) to 5 (fully disagree). We reversed scored these responses so a higher score on the derived construct indicates higher level of perception that peers are carrying out the behaviors prompted about. In the single-country-CFAs and in the assessment of measurement invariance we removed all respondents that answered “don’t know” in one or more of the four questions. Following that exclusion criterion, there were 1208 remaining respondents which were used in the analyses reported below. After carrying out the analyses we assigned factor scores to the 1208 respondent that answered all four items, and to the 242 respondents that answered “Don’t know” to one question. The remaining 208 respondents that answered “Don’t know” two or more times were not assigned a score, as we speculated that we had too little information available about them to assign a score on the latent continuum.

Table 1. Overview of items used to measure Peer perceptions.

	COMMON INTRODUCTORY QUESTION:<br>To what extent do you agree with the following statements?<br>It is common for my classmates to...
	**Statement**
Item 1	delete data from an experiment only because it somehow seemed wrong.
Item 2	give a misleading or dubious interpretation of texts, works of art or interview data in order to achieve results the teacher will accept.
Item 3	receive help from other students or family members on assignments they were supposed to complete on their own.
Item 4	copy shorter passages from other sources into their own texts without marking them as quotes.

All questions were measures on the response scale: 1 (Fully agree) to 5 (Fully disagree) plus a “Don’t know” option.

Following Davidov (2009[1]) we started out by running country-specific CFA prior to estimation of measurement invariance. As described in the main text we evaluate whether the single-country models are well-fitting using root mean square error of approximation (RMSEA), standardized root mean squared residual (SRMR), and the comparative fit index (CFI). Stata’s *sem* command was used for the analyses. **Table 2** give an overview of the single-country confirmatory factor analyses (CFA). It is seen that the model fit in all countries are acceptable. In Denmark and Ireland, it was necessary to allow item 3 (“receive help from other students or family members on assignments they were supposed to complete on their own”) and item 4 (“copy shorter passages from other sources into their own texts without marking them as quotes”) to co-vary (because of error correlation). Both actions (Receiving help from others and Copying shorter passages) are examples where external sources are used to carry out the questionable behavior. So it makes sense that these two items can be correlated.

[1] Davidov E. 2009. Measurement equivalence of nationalism and constructive patriotism in the ISSP: 34 countries in a comparative perspective. Polit. Anal. 17(1):64–82

Table 2. Country-specific results from CFA with global fit indices and model modifications.

	Modification	CFI	SRMR	RMSEA	PCLOSE
Denmark (n=275)	Item 3 & 4 (correlated errors)	1.00	0.005	0.000	0.773
Ireland (n=194)	Item 3 & 4 (correlated errors)	0.99	0.016	0.050	0.329
Lithuania (n=131)		1.00	0.025	0.000	0.506
Portugal (n=166)		1.00	0.016	0.000	0.693
Switzerland (French speaking) (n=271)		1.00	0.007	0.000	0.934
Slovenia (n=171)		1.00	0.024	0.000	0.614

Then we followed up with multi-group CFA to test measurement invariance (**Table 3**). We first tried out the configural model. This model exhibited a relatively good fit. But the RMSEA exceed 0.05. Therefore, we allowed item 3 and item 4 to co-vary. As mentioned above, it is justified to assume that these two items can be correlated because they are both examples of the use of external sources. The resulting configural model was acceptable. Further, the metric model (where we also allowed correlated errors between item 3 and 4) also showed a good fit. Finally, the last row of Table 3 shows that we did not identify scalar invariance.

Table 3. Country-specific results from CFA with global fit indices and model modifications (n=1208)

	Modification	CFI	SRMR	RMSEA
CONFIGURAL (v1)		0.986	0.030	0.058
CONFIGURAL (v2)	Item 3 & 4 (correlated errors)	1.00	0.012	0.000
METRIC	Item 3 & 4 (correlated errors)	1.00	0.057	0.000
SCALAR	Item 3 & 4 (correlated errors)	0.866	0.062	0.092

We calculated factor scores after the metric invariance model was run using the *predict* command in Stata. A histogram of the derived Peer Perception construct can be seen in the Figure below.

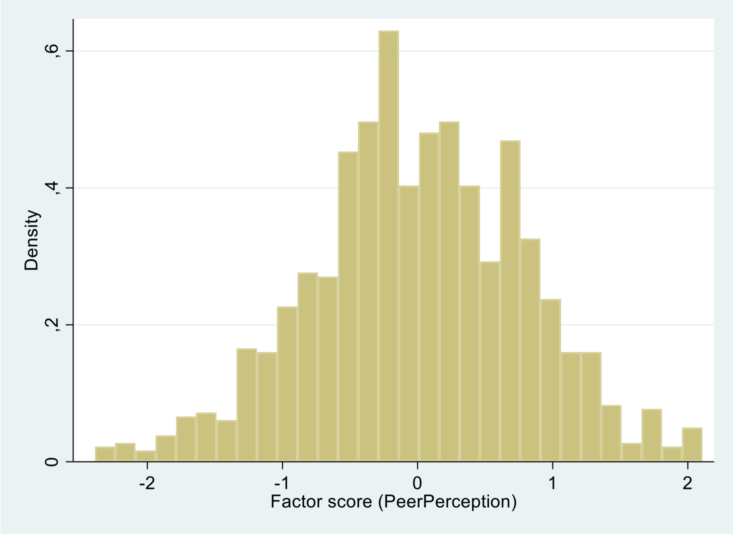

Descriptive summary on the 1208 respondents that answered all four items (from Table 1) on the 1 to 5 response scale are:

```
    Variable   |        Obs         Mean    Std. dev.       Min        Max
---------------+-----------------------------------------------------------
PeerPerception |      1.208    -2,07e-10    ,7960122  -2,387421   2,108576
```

Descriptive summary of the 1450 respondents that either answered all four items (from Table 1) on the 1 to 5 response scale or answered three of the items on the 1 to 5 response scale, and responded don't know" to a fourth item were:

Variable	Obs	Mean	Std. dev.	Min	Max
PeerPerception	1.450	-,0200897	,796188	-2,387421	2,108576

On the next three pages, we provide the Stata output from the single country CFAs (i.e. country-specific (unstandardized) factor loadings) whose global model fits were reported in Table 2. Following that, we give the Stata output from the metric invariance model.

Country-specific (unstandardized) factor loadings

DENMARK

	Coefficient	OIM std. err.	z	P>|z|	[95% conf.	interval]
Measurement						
item_1						
PeerPerception	,6372092	,0833419	7,65	0,000	,4738621	,8005563
_cons	2,687273	,0687172	39,11	0,000	2,552589	2,821956
item_2						
PeerPerception	,8477463	,0898074	9,44	0,000	,671727	1,023766
_cons	2,843636	,0642594	44,25	0,000	2,71769	2,969583
item_3						
PeerPerception	,3082524	,0721962	4,27	0,000	,1667504	,4497544
_cons	3,727273	,0593681	62,78	0,000	3,610913	3,843632
item_4						
PeerPerception	,5894289	,0826857	7,13	0,000	,4273679	,7514898
_cons	3,072727	,0692386	44,38	0,000	2,937022	3,208433
var(e.item_1)	,8925297	,1029773			,7118913	1,119004
var(e.item_2)	,4168766	,1278095			,2285804	,7602844
var(e.item_3)	,8742366	,0791556			,7320809	1,043996
var(e.item_4)	,9709207	,1028243			,7889295	1,194894
var(PeerPerception)	1	(constrained)				
cov(e.item_3,e.item_4)	,1508691	,0663077	2,28	0,023	,0209083	,2808299

LR test of model vs. saturated: chi2(1) = 0,16 Prob > chi2 = 0,6854

IRELAND

	Coefficient	OIM std. err.	z	P>|z|	[95% conf.	interval]
Measurement						
item_1						
PeerPerception	,5761854	,0927836	6,21	0,000	,3943329	,7580379
_cons	3,128866	,0771094	40,58	0,000	2,977734	3,279998
item_2						
PeerPerception	,7134328	,0984848	7,24	0,000	,5204061	,9064595
_cons	3,170103	,074944	42,30	0,000	3,023216	3,316991
item_3						
PeerPerception	,4706373	,0945431	4,98	0,000	,2853363	,6559383
_cons	3,778351	,0735271	51,39	0,000	3,63424	3,922461
item_4						
PeerPerception	,7197424	,1053746	6,83	0,000	,5132121	,9262728
_cons	3,634021	,0819921	44,32	0,000	3,473319	3,794722
var(e.item_1)	,8215067	,1078423			,6351417	1,062555
var(e.item_2)	,5806351	,1202434			,3869264	,8713209
var(e.item_3)	,8273102	,1033991			,6475648	1,056948
var(e.item_4)	,7861743	,1349687			,5615473	1,100655
var(PeerPerception)	1	(constrained)				
cov(e.item_3,e.item_4)	,3327206	,0953797	3,49	0,000	,1457798	,5196613

LR test of model vs. saturated: chi2(1) = 1,48 Prob > chi2 = 0,2235

LITHUANIA

	Coefficient	OIM std. err.	z	P>|z|	[95% conf.	interval]
Measurement						
item_1						
PeerPerception	,5077224	,1046627	4,85	0,000	,3025872	,7128575
_cons	3,045802	,0902331	33,75	0,000	2,868948	3,222655
item_2						
PeerPerception	,5851949	,1198431	4,88	0,000	,3503067	,8200831
_cons	2,908397	,1032373	28,17	0,000	2,706056	3,110738
item_3						
PeerPerception	,6781791	,1014981	6,68	0,000	,4792464	,8771118
_cons	3,755725	,0877641	42,79	0,000	3,583711	3,92774
item_4						
PeerPerception	,7656249	,115662	6,62	0,000	,5389315	,9923183
_cons	3,541985	,1002645	35,33	0,000	3,34547	3,7385
var(e.item_1)	,8088225	,1179068			,607811	1,076311
var(e.item_2)	1,053736	,1543272			,790801	1,404095
var(e.item_3)	,5491052	,1123158			,3677461	,8199042
var(e.item_4)	,7307581	,1455826			,494534	1,079819
var(PeerPerception)	1	(constrained)				

LR test of model vs. saturated: chi2(2) = 1,85 Prob > chi2 = 0,39

PORTUGAL

	Coefficient	OIM std. err.	z	P>|z|	[95% conf.	interval]
Measurement						
item_1						
PeerPerception	,5750222	,097137	5,92	0,000	,3846371	,7654072
_cons	3,018072	,0841102	35,88	0,000	2,853219	3,182925
item_2						
PeerPerception	,589794	,0929058	6,35	0,000	,4077019	,7718861
_cons	3,168675	,079298	39,96	0,000	3,013253	3,324096
item_3						
PeerPerception	,4742862	,0816737	5,81	0,000	,3142086	,6343638
_cons	3,746988	,0711184	52,69	0,000	3,607598	3,886377
item_4						
PeerPerception	,8214785	,1077285	7,63	0,000	,6103346	1,032622
_cons	3,355422	,0905681	37,05	0,000	3,177912	3,532932
var(e.item_1)	,8437216	,1140906			,6472873	1,099769
var(e.item_2)	,6959809	,1027349			,5211349	,9294897
var(e.item_3)	,6146519	,0813178			,4742597	,7966036
var(e.item_4)	,6868004	,1426712			,4570964	1,031937
var(PeerPerception)	1	(constrained)				

LR test of model vs. saturated: chi2(2) = 1,09 Prob > chi2 = 0,5807

SWITZERLAND(FRENCH SPEAKING)

	Coefficient	OIM std. err.	z	P>|z|	[95% conf.	interval]
Measurement						
item_1						
PeerPerception	,5599762	,0884964	6,33	0,000	,3865265	,7334259
_cons	3,095941	,0699654	44,25	0,000	2,958811	3,233071
item_2						
PeerPerception	,6504372	,0890828	7,30	0,000	,4758382	,8250363
_cons	3,162362	,0691186	45,75	0,000	3,026892	3,297832
item_3						
PeerPerception	,4952453	,0742265	6,67	0,000	,3497641	,6407266
_cons	4,0369	,0585346	68,97	0,000	3,922175	4,151626
item_4						
PeerPerception	,6674681	,0949456	7,03	0,000	,4813781	,8535582
_cons	3,191882	,0741709	43,03	0,000	3,04651	3,337254
var(e.item_1)	1,013015	,1094636			,8196673	1,251971
var(e.item_2)	,8716033	,1107805			,6794092	1,118166
var(e.item_3)	,6832597	,0770269			,5478051	,8522078
var(e.item_4)	1,045343	,1256596			,8259176	1,323064
var(PeerPerception)	1	(constrained)				

LR test of model vs. saturated: chi2(2) = 0,26 Prob > chi2 = 0,8762

SLOVENIA

	Coefficient	OIM std. err.	z	P>|z|	[95% conf.	interval]
Measurement						
item_1						
PeerPerception	,546576	,1198445	4,56	0,000	,3116852	,7814669
_cons	2,842105	,082441	34,47	0,000	2,680524	3,003687
item_2						
PeerPerception	,7299821	,1432794	5,09	0,000	,4491597	1,010805
_cons	2,842105	,0820251	34,65	0,000	2,681339	3,002871
item_3						
PeerPerception	,2207329	,099133	2,23	0,026	,0264358	,41503
_cons	3,643275	,0728627	50,00	0,000	3,500467	3,786083
item_4						
PeerPerception	,4129436	,1113181	3,71	0,000	,1947641	,6311231
_cons	3,350877	,0833097	40,22	0,000	3,187593	3,514161
var(e.item_1)	,8634586	,1371324			,632493	1,178765
var(e.item_2)	,6176338	,1928711			,3349059	1,139041
var(e.item_3)	,8591119	,097677			,6874991	1,073563
var(e.item_4)	1,016304	,1270382			,7954694	1,298446
var(PeerPerception)	1	(constrained)				

LR test of model vs. saturated: chi2(2) = 1,46 Prob > chi2 = 0,4830

METRIC INVARIANCE MODEL OUTPUT (unstandardized coefficients)

Structural equation model Number of obs = 1.208
Grouping variable: Country Number of groups = 6
Estimation method: mlmv

Group: Denmark Number of obs = 275

	Coefficient	OIM std. err.	z	P>|z|	[95% conf.	interval]
**Measurement**						
item_1						
PeerPerception	,5880545	,0399995	14,70	0,000	,5096569	,6664521
_cons	2,687273	,0671279	40,03	0,000	2,555705	2,818841
item_2						
PeerPerception	,7143983	,0430514	16,59	0,000	,6300191	,7987774
_cons	2,843636	,0621318	45,77	0,000	2,72186	2,965412
item_3						
PeerPerception	,4002428	,0376936	10,62	0,000	,3263647	,474121
_cons	3,727273	,0602611	61,85	0,000	3,609163	3,845382
item_4						
PeerPerception	,6276067	,04387	14,31	0,000	,5416231	,7135902
_cons	3,072727	,0689363	44,57	0,000	2,937615	3,20784
mean(PeerPerception)	0	(constrained)				
var(e.item_1)	,893383	,0914602			,7309636	1,091892
var(e.item_2)	,5512327	,0819173			,4119458	,7376153
var(e.item_3)	,8384397	,0785401			,6978085	1,007413
var(e.item_4)	,9129689	,097077			,7412194	1,124515
var(PeerPerception)	1	(constrained)				
cov(e.item_3,e.item_4)	,1007369	,0638775	1,58	0,115	-,0244607	,2259346

Group: Ireland Number of obs = 194

	Coefficient	OIM std. err.	z	P>|z|	[95% conf.	interval]
**Measurement**						
item_1						
PeerPerception	,5880545	,0399995	14,70	0,000	,5096569	,6664521
_cons	3,128866	,0770915	40,59	0,000	2,977769	3,279963
item_2						
PeerPerception	,7143983	,0430514	16,59	0,000	,6300191	,7987774
_cons	3,170103	,0742371	42,70	0,000	3,024601	3,315605
item_3						
PeerPerception	,4002428	,0376936	10,62	0,000	,3263647	,474121
_cons	3,778351	,07246	52,14	0,000	3,636332	3,920369
item_4						
PeerPerception	,6276067	,04387	14,31	0,000	,5416231	,7135902
_cons	3,634021	,0800443	45,40	0,000	3,477137	3,790904
mean(PeerPerception)	0	(constrained)				
var(e.item_1)	,8071544	,1000625			,6330435	1,029152
var(e.item_2)	,558797	,0935223			,4025259	,7757367
var(e.item_3)	,8583923	,0955781			,6900942	1,067734
var(e.item_4)	,8490838	,1086879			,6606806	1,091213
var(PeerPerception)	1	(constrained)				
cov(e.item_3,e.item_4)	,3770433	,080456	4,69	0,000	,2193525	,5347342

Group: Lithuania Number of obs = 131

	Coefficient	OIM std. err.	z	P>|z|	[95% conf. interval]	
**Measurement**						
item_1						
PeerPerception	,5880545	,0399995	14,70	0,000	,5096569	,6664521
_cons	3,045802	,090978	33,48	0,000	2,867488	3,224115
item_2						
PeerPerception	,7143983	,0430514	16,59	0,000	,6300191	,7987774
_cons	2,908397	,1039088	27,99	0,000	2,704739	3,112054
item_3						
PeerPerception	,4002428	,0376936	10,62	0,000	,3263647	,474121
_cons	3,755725	,0850457	44,16	0,000	3,589039	3,922412
item_4						
PeerPerception	,6276067	,04387	14,31	0,000	,5416231	,7135902
_cons	3,541985	,1001852	35,35	0,000	3,345625	3,738344
mean(PeerPerception)	0	(constrained)				
var(e.item_1)	,7384774	,1152788			,5438283	1,002796
var(e.item_2)	,9040477	,1491353			,6542952	1,249134
var(e.item_3)	,7872988	,1092804			,5997762	1,033451
var(e.item_4)	,9209664	,1415389			,6814387	1,244689
var(PeerPerception)	1	(constrained)				
cov(e.item_3,e.item_4)	,2368655	,0940959	2,52	0,012	,052441	,42129

Group: Portugal Number of obs = 166

	Coefficient	OIM std. err.	z	P>|z|	[95% conf. interval]	
**Measurement**						
item_1						
PeerPerception	,5880545	,0399995	14,70	0,000	,5096569	,6664521
_cons	3,018072	,0840634	35,90	0,000	2,853311	3,182834
item_2						
PeerPerception	,7143983	,0430514	16,59	0,000	,6300191	,7987774
_cons	3,168675	,0806477	39,29	0,000	3,010608	3,326741
item_3						
PeerPerception	,4002428	,0376936	10,62	0,000	,3263647	,474121
_cons	3,746988	,0709971	52,78	0,000	3,607836	3,88614
item_4						
PeerPerception	,6276067	,04387	14,31	0,000	,5416231	,7135902
_cons	3,355422	,0884688	37,93	0,000	3,182026	3,528817
mean(PeerPerception)	0	(constrained)				
var(e.item_1)	,8272564	,1095809			,638098	1,072489
var(e.item_2)	,5693088	,0987323			,4052551	,7997742
var(e.item_3)	,6765441	,0833743			,531371	,8613792
var(e.item_4)	,9053467	,1236813			,6926767	1,183312
var(PeerPerception)	1	(constrained)				
cov(e.item_3,e.item_4)	,1358709	,0752032	1,81	0,071	-,0115247	,2832665

Group: Switz_French Number of obs = 271

	Coefficient	OIM std. err.	z	P>|z|	[95% conf. interval]	
Measurement						
item_1						
PeerPerception	,5880545	,0399995	14,70	0,000	,5096569	,6664521
_cons	3,095941	,0704307	43,96	0,000	2,957899	3,233983
item_2						
PeerPerception	,7143983	,0430514	16,59	0,000	,6300191	,7987774
_cons	3,162362	,0698481	45,27	0,000	3,025462	3,299261
item_3						
PeerPerception	,4002428	,0376936	10,62	0,000	,3263647	,474121
_cons	4,0369	,0575492	70,15	0,000	3,924106	4,149695
item_4						
PeerPerception	,6276067	,04387	14,31	0,000	,5416231	,7135902
_cons	3,191882	,0739151	43,18	0,000	3,047011	3,336753
mean(PeerPerception)	0	(constrained)				
var(e.item_1)	,998482	,1024942			,8165155	1,221001
var(e.item_2)	,8117771	,1008019			,6364135	1,035462
var(e.item_3)	,737333	,0730086			,6072677	,895256
var(e.item_4)	1,086703	,1158054			,8818648	1,339121
var(PeerPerception)	1	(constrained)				
cov(e.item_3,e.item_4)	,0388698	,0668883	0,58	0,561	-,0922288	,1699684

Group: Slovenia Number of obs = 171

	Coefficient	OIM std. err.	z	P>|z|	[95% conf. interval]	
Measurement						
item_1						
PeerPerception	,5880545	,0399995	14,70	0,000	,5096569	,6664521
_cons	2,842105	,0843403	33,70	0,000	2,676801	3,007409
item_2						
PeerPerception	,7143983	,0430514	16,59	0,000	,6300191	,7987774
_cons	2,842105	,084639	33,58	0,000	2,676216	3,007995
item_3						
PeerPerception	,4002428	,0376936	10,62	0,000	,3263647	,474121
_cons	3,643275	,0760019	47,94	0,000	3,494314	3,792236
item_4						
PeerPerception	,6276067	,04387	14,31	0,000	,5416231	,7135902
_cons	3,350877	,0882791	37,96	0,000	3,177853	3,523901
mean(PeerPerception)	0	(constrained)				
var(e.item_1)	,8705648	,1128856			,6751905	1,122473
var(e.item_2)	,7146378	,1129381			,5242832	,9741055
var(e.item_3)	,8275515	,0986282			,6551613	1,045302
var(e.item_4)	,9387478	,1249844			,7231367	1,218646
var(PeerPerception)	1	(constrained)				
cov(e.item_3,e.item_4)	,0123582	,0799848	0,15	0,877	-,1444091	,1691255

LR test of model vs. saturated: chi2(26) = 22,84 Prob > chi2 = 0,6418
