# Supplementary material for: Lack of ethics or lack of knowledge? European upper secondary students’ doubts and misconceptions about integrity issues
Source: Int J Educ Integr. 2022 Aug 11;18(1):20. doi: 10.1007/s40979-022-00113-0 (PMC9365441; doi:10.1007/s40979-022-00113-0)
Supplement: Supplementary file 8 — Additional file 8. Assessment of measurement invariance of self-reported knowledge [file 40979_2022_113_MOESM8_ESM.pdf]

Additional file 8: Assessment of measurement invariance of self-reported knowledge

Table 1 shows the six questions that were used as input variables (manifest variables) to the confirmatory factor analysis (CFA). Response options ranged from 1 (fully agree) to 5 (fully disagree). We reversed scored these responses so a higher score on the derived construct indicates higher level of self-reported knowledge. In the single-country CFAs and in the assessment of measurement invariance we removed all respondents that answered "don't know" to one or more of the four questions. This means that 1076 respondents were used in the CFA analyses reported below. After carrying out the CFA we assigned factor scores to all 1654. In the subsequent regression models (see Additional file 9) we only included respondents that had given responses to both dimension-specific questions. For instance, for the questionable behavior "Have you…deleted data from an experiment only because it somehow seemed wrong" respondents were excluded from the analysis if they did not give a respond ranging between 1 and 5 on item 3 and item 6.

Table 1. Overview of items used to measure Self-reported knowledge.

	COMMON INTRODUCTORY QUESTION AND INSTRUCTION TO ITEM 1 to 3:<br>To what extent do you agree with the following claim:<br>I have a good understanding of the official standards of good practice that apply to me in relation to...<br>(Examples of "official standards of good practice" could be rules and regulations stating what is prohibited, but it could also include guidelines and codes of conduct describing how to behave correctly.)
	**Statement**
Item 1	citation and plagiarism
Item 2	working with others and assigning authorship
Item 3	collection, analysis and presentation of data
	COMMON INTRODUCTORY QUESTION TO ITEM 4 to 6:<br>To what extent do you agree with the following claim:<br>In general, I know how to behave in an ethically correct manner in relation to ...
	**Statement**
Item 4	citation and plagiarism
Item 5	working with others and assigning authorship
Item 6	collection, analysis and presentation of data

All questions were measured on the response scale: 1 (Fully agree) to 5 (Fully disagree) plus a "Don't know" option.

Following Davidov (Davidov 2009[1]) we started out by running country-specific CFA prior to estimation of measurement invariance. As described in the main text we evaluate whether the single-country models are well-fitting using root mean square error of approximation (RMSEA), standardized root mean squared residual (SRMR), and the comparative fit index (CFI). Stata's *sem* command was used for the analyses. **Table 2** give an overview of the single-country confirmatory factor analyses (CFA). It can be seen there is poor model fit in all countries. It was not possible to improve the fit to to the required level through modification.

[1] Davidov E. 2009. Measurement equivalence of nationalism and constructive patriotism in the ISSP: 34 countries in a comparative perspective. Polit. Anal. 17(1):64–82

Table 2. Country-specific results from CFA with global fit indices and model modifications.

	Modification	CFI	SRMR	RMSEA	PCLOSE
Denmark (n=256)		0.792	0.080	0.178	0.000
Ireland (n=200)		0.866	0.069	0.050	0.000
Lithuania (n=133)		0.855	0.084	0.184	0.000
Portugal (n=161)		0.592	0.220	0.434	0.000
Switzerland (French speaking) (n=200)		0.612	0.136	0.321	0.000
Slovenia (n=171)		0.688	0.100	0.260	0.000

Since the least restrictive model was rejected there was then no reason to follow up with multi-group CFA to test measurement invariance.

In the table (**Table 3**) below we report the country-specific internal consistency coefficients (Cronbach's alpha).

Table 3. Country-specific internal consistency coefficients

Country	Cronbach's alpha	Proportion of explained variance (first component)
Denmark (n=256)	0.81	52%
Ireland (n=200)	0.82	54%
Lithuania (n=133)	0.81	53%
Portugal (n=161)	0.80	52%
Switzerland (French speaking) (n=200)	0.80	49%
Slovenia (n=171)	0.80	49%
All countries (n=1076)	0.80	50%

The coefficients are acceptable in all countries. So even though the construct is not country invariant at an acceptable level it does tap into self-reported knowledge.

We then ran a CFA model with all countries pooled. We did this to calculate factor scores (using the *predict* command in Stata) so that the Self-reported Knowledge construct could be used in subsequent analysis. A histogram of the derived Self-reported Knowledge construct can be seen in the Figure below.

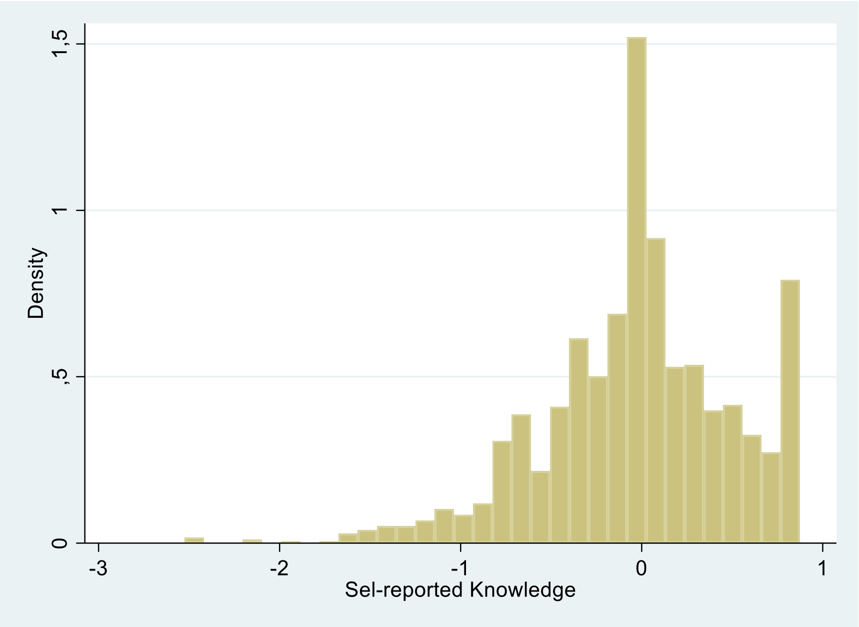

Here is a descriptive summary on the 1076 respondents that answered all six items (from Table 1) on the 1 to 5 response scale

Variable	Obs	Mean	Std. dev.	Min	Max
Self_Knowledge	1.076	2,86e-09	,5329727	-2,523328	,8743362

On the next three pages, we provide the Stata output from the single country CFAs (i.e. country-specific (unstandardized) factor loadings) whose global model fits were reported in Table 2. Following that, we give the Stata output from the pooled CFA (where all countries were included) used to calculate factor scores.

Country-specific (unstandardized) factor loadings

DENMARK

	Coefficient	OIM std. err.	z	P>|z|	[95% conf.	interval]
Measurement						
item_1						
Self_Knowledge	1	(constrained)				
_cons	3,964844	,0501286	79,09	0,000	3,866594	4,063094
item_2						
Self_Knowledge	1,161143	,1758163	6,60	0,000	,8165492	1,505737
_cons	3,769531	,0496266	75,96	0,000	3,672265	3,866798
item_3						
Self_Knowledge	,9305498	,1370894	6,79	0,000	,6618595	1,19924
_cons	4,023438	,039424	102,06	0,000	3,946168	4,100707
item_4						
Self_Knowledge	1,234442	,173544	7,11	0,000	,8943023	1,574582
_cons	4,101563	,0486888	84,24	0,000	4,006134	4,196991
item_5						
Self_Knowledge	1,473938	,2044551	7,21	0,000	1,073213	1,874662
_cons	3,992188	,049716	80,30	0,000	3,894746	4,089629
item_6						
Self_Knowledge	1,288247	,1761241	7,31	0,000	,9430497	1,633444
_cons	4,050781	,043206	93,76	0,000	3,966099	4,135463
var(e.item_1)	,4740022	,0457495			,3923057	,5727118
var(e.item_2)	,402228	,0422697			,3273561	,4942245
var(e.item_3)	,2512934	,0256031			,2058052	,3068357
var(e.item_4)	,3488957	,0372817			,2829694	,4301815
var(e.item_5)	,2649636	,0338567			,2062628	,3403702
var(e.item_6)	,1969347	,0256707			,1525341	,2542596
var(Self_Knowledge)	,1692931	,0426081			,1033728	,2772502

LR test of model vs. saturated: chi2(9) = 123,24 Prob > chi2 = 0,0000

IRELAND

	Coefficient	OIM std. err.	z	P>|z|	[95% conf.	interval]
Measurement						
item_1						
Self_Knowledge	1	(constrained)				
_cons	3,685	,0743563	49,56	0,000	3,539264	3,830736
item_2						
Self_Knowledge	,603905	,0878336	6,88	0,000	,4317542	,7760557
_cons	4,1	,0533854	76,80	0,000	3,995367	4,204633
item_3						
Self_Knowledge	,5152835	,0878521	5,87	0,000	,3430966	,6874704
_cons	4,025	,0526486	76,45	0,000	3,921811	4,128189
item_4						
Self_Knowledge	1,101384	,107749	10,22	0,000	,8901996	1,312568
_cons	3,77	,070608	53,39	0,000	3,631611	3,908389
item_5						
Self_Knowledge	,7263002	,0974335	7,45	0,000	,5353341	,9172663
_cons	4,095	,0559453	73,20	0,000	3,985349	4,204651
item_6						
Self_Knowledge	,7306554	,0933787	7,82	0,000	,5476366	,9136743
_cons	4,065	,0524773	77,46	0,000	3,962146	4,167854
var(e.item_1)	,5740118	,0757814			,4431438	,7435275
var(e.item_2)	,376066	,042218			,301791	,4686212
var(e.item_3)	,4131833	,0451239			,3335667	,511803
var(e.item_4)	,352049	,06124			,2503422	,4950764
var(e.item_5)	,3454646	,0424104			,2715859	,4394403
var(e.item_6)	,2668903	,0359073			,2050277	,3474186
var(Self_Knowledge)	,5317598	,1066782			,3588831	,7879126

LR test of model vs. saturated: chi2(9) = 65,66 Prob > chi2 = 0,0000

LITHUANIA

	Coefficient	OIM std. err.	z	P>|z|	[95% conf.	interval]
Measurement						
item_1						
Self_Knowledge	1	(constrained)				
_cons	3,781955	,0879014	43,02	0,000	3,609671	3,954238
item_2						
Self_Knowledge	,7003907	,1774385	3,95	0,000	,3526176	1,048164
_cons	4,007519	,0684964	58,51	0,000	3,873268	4,141769
item_3						
Self_Knowledge	,9391936	,1945748	4,83	0,000	,557834	1,320553
_cons	4,165414	,0621659	67,00	0,000	4,043571	4,287256
item_4						
Self_Knowledge	1,325143	,254233	5,21	0,000	,8268557	1,823431
_cons	3,947368	,0731419	53,97	0,000	3,804013	4,090724
item_5						
Self_Knowledge	1,209724	,241571	5,01	0,000	,7362537	1,683195
_cons	4,090226	,0684654	59,74	0,000	3,956036	4,224415
item_6						
Self_Knowledge	1,218331	,2390793	5,10	0,000	,7497446	1,686918
_cons	4,090226	,0650788	62,85	0,000	3,962674	4,217778
var(e.item_1)	,7881815	,1032626			,6096873	1,018932
var(e.item_2)	,5065358	,0654177			,39326	,6524399
var(e.item_3)	,3027653	,0426479			,2297232	,3990316
var(e.item_4)	,2910179	,0486668			,209688	,4038924
var(e.item_5)	,2730006	,0439345			,199149	,374239
var(e.item_6)	,2078457	,0372848			,1462334	,2954171
var(Self_Knowledge)	,2394628	,0886569			,1159033	,4947439

LR test of model vs. saturated: chi2(9) = 49,06 Prob > chi2 = 0,0000

.

PORTUGAL

	Coefficient	OIM std. err.	z	P>|z|	[95% conf.	interval]
Measurement						
item_1						
Self_Knowledge	1	(constrained)				
_cons	3,248447	,1016932	31,94	0,000	3,049132	3,447762
item_2						
Self_Knowledge	,4536178	,0585085	7,75	0,000	,3389433	,5682923
_cons	3,888199	,0805031	48,30	0,000	3,730416	4,045982
item_3						
Self_Knowledge	,0877007	,0488507	1,80	0,073	-,0080449	,1834463
_cons	4,21118	,0585368	71,94	0,000	4,09645	4,32591
item_4						
Self_Knowledge	,9785496	,0419744	23,31	0,000	,8962814	1,060818
_cons	3,304348	,0966406	34,19	0,000	3,114936	3,49376
item_5						
Self_Knowledge	,4105352	,0591192	6,94	0,000	,2946637	,5264067
_cons	3,881988	,0792401	48,99	0,000	3,72668	4,037295
item_6						
Self_Knowledge	,1482889	,0558594	2,65	0,008	,0388066	,2577713
_cons	4,10559	,0678143	60,54	0,000	3,972676	4,238504
var(e.item_1)	,1854037	,0442425			,1161443	,295964
var(e.item_2)	,7389496	,0842252			,5910104	,9239202
var(e.item_3)	,5402962	,0602767			,4341801	,6723477
var(e.item_4)	,0868617	,0406499			,034712	,217359
var(e.item_5)	,7615509	,0862972			,6098777	,9509445
var(e.item_6)	,7078682	,0790531			,5687116	,8810746
var(Self_Knowledge)	1,479578	,1885211			1,152608	1,899303

LR test of model vs. saturated: chi2(9) = 279,84 Prob > chi2 = 0,0000

SWITZERLAND(FRENCH SPEAKING)

	Coefficient	OIM std. err.	z	P>|z|	[95% conf.	interval]
Measurement						
item_1						
Self_Knowledge	1	(constrained)				
_cons	3,965	,075953	52,20	0,000	3,816135	4,113865
item_2						
Self_Knowledge	1,463788	,3222941	4,54	0,000	,8321027	2,095473
_cons	4,085	,0706319	57,84	0,000	3,946564	4,223436
item_3						
Self_Knowledge	1,736486	,3814154	4,55	0,000	,9889255	2,484047
_cons	4,07	,0606257	67,13	0,000	3,951176	4,188824
item_4						
Self_Knowledge	,9880238	,2425749	4,07	0,000	,5125857	1,463462
_cons	4,025	,0694397	57,96	0,000	3,888901	4,161099
item_5						
Self_Knowledge	1,510841	,333936	4,52	0,000	,8563384	2,165343
_cons	4,03	,0713827	56,46	0,000	3,890093	4,169907
item_6						
Self_Knowledge	1,668296	,369216	4,52	0,000	,9446458	2,391946
_cons	4,075	,0599738	67,95	0,000	3,957454	4,192546
var(e.item_1)	,9911958	,1042425			,806566	1,218089
var(e.item_2)	,6494236	,0785975			,5122832	,823277
var(e.item_3)	,244865	,0461671			,1692156	,3543342
var(e.item_4)	,805668	,0854199			,6544984	,9917533
var(e.item_5)	,6479932	,0802591			,508326	,8260353
var(e.item_6)	,2668861	,0446767			,1922362	,3705245
var(Self_Knowledge)	,1625765	,0672931			,0722323	,3659183

LR test of model vs. saturated: chi2(9) = 193,45 Prob > chi2 = 0,0000

SLOVENIA

	Coefficient	OIM std. err.	z	P>|z|	[95% conf.	interval]
Measurement						
item_1						
Self_Knowledge	1	(constrained)				
_cons	3,769841	,0870579	43,30	0,000	3,599211	3,940472
item_2						
Self_Knowledge	1,177028	,2713285	4,34	0,000	,6452337	1,708822
_cons	3,753968	,0845054	44,42	0,000	3,588341	3,919596
item_3						
Self_Knowledge	1,007674	,21344	4,72	0,000	,5893388	1,426008
_cons	4	,0634921	63,00	0,000	3,875558	4,124442
item_4						
Self_Knowledge	1,095106	,2303763	4,75	0,000	,6435763	1,546635
_cons	3,896825	,0823482	47,32	0,000	3,735426	4,058225
item_5						
Self_Knowledge	1,142515	,2571146	4,44	0,000	,6385796	1,64645
_cons	3,896825	,0768075	50,73	0,000	3,746286	4,047365
item_6						
Self_Knowledge	,8504111	,1966002	4,33	0,000	,4650818	1,23574
_cons	3,952381	,062348	63,39	0,000	3,830181	4,074581
var(e.item_1)	,704193	,1001913			,5328246	,9306772
var(e.item_2)	,55237	,0888999			,4029354	,7572246
var(e.item_3)	,2533028	,0464216			,176866	,3627735
var(e.item_4)	,5536964	,0855125			,4090843	,7494292
var(e.item_5)	,4159826	,0708826			,2978736	,5809226
var(e.item_6)	,3084391	,0478016			,2276413	,4179148
var(Self_Knowledge)	,2507702	,0936549			,1206086	,5214029

LR test of model vs. saturated: chi2(9) = 84,80 Prob > chi2 = 0,0000

POOLED CFA (ALL COUNTRIES) (unstandardized coefficients)

```
Structural equation model                         Number of obs = 1.076
Estimation method: ml

Log likelihood = -7533,9183

 ( 1)  [item_1]Self_Knowledge = 1
```

	Coefficient	OIM std. err.	z	P>|z|	[95% conf.	interval]
Measurement						
item_1						
Self_Knowledge	1	(constrained)				
_cons	3,760223	,0322497	116,60	0,000	3,697015	3,823431
item_2						
Self_Knowledge	,9808563	,0701427	13,98	0,000	,8433791	1,118333
_cons	3,934944	,0272773	144,26	0,000	3,881482	3,988407
item_3						
Self_Knowledge	,7355934	,0574973	12,79	0,000	,6229007	,848286
_cons	4,075279	,0225149	181,00	0,000	4,03115	4,119407
item_4						
Self_Knowledge	1,055847	,0661465	15,96	0,000	,926202	1,185491
_cons	3,863383	,0303281	127,39	0,000	3,803941	3,922825
item_5						
Self_Knowledge	1,092724	,0757443	14,43	0,000	,9442675	1,24118
_cons	4,002788	,0268874	148,87	0,000	3,95009	4,055486
item_6						
Self_Knowledge	,8500897	,0623896	13,63	0,000	,7278084	,972371
_cons	4,05948	,0234045	173,45	0,000	4,013608	4,105351
var(e.item_1)	,7689683	,0398906			,6946275	,8512653
var(e.item_2)	,4637587	,0251562			,4169839	,5157804
var(e.item_3)	,356	,0183488			,3217939	,3938422
var(e.item_4)	,5993836	,0331772			,5377605	,6680682
var(e.item_5)	,3598159	,0225951			,3181471	,4069421
var(e.item_6)	,3363849	,0186165			,3018065	,374925
var(Self_Knowledge)	,3501186	,0414834			,277563	,4416404

```
LR test of model vs. saturated: chi2(9) = 787,26            Prob > chi2 = 0,0000
```
