# Supplementary material for: Lack of ethics or lack of knowledge? European upper secondary students’ doubts and misconceptions about integrity issues
Source: Int J Educ Integr. 2022 Aug 11;18(1):20. doi: 10.1007/s40979-022-00113-0 (PMC9365441; doi:10.1007/s40979-022-00113-0)
Supplement: Supplementary file 9 — Additional file 9. Output from the four regression models [file 40979_2022_113_MOESM9_ESM.pdf]

Additional file 9: Output from the four regression models

Regression results[1] from outcome variable

Deleted deviating data points based on a gut feeling that they were inaccurate (0=no; 1=yes)

```
Mixed-effects logistic regression               Number of obs     =        902
Group variable: HighSchools                     Number of groups  =         51

                                                Obs per group:
                                                              min =          2
                                                              avg =       17,7
                                                              max =         84

Integration method: mvaghermite                 Integration pts.  =          7

                                                Wald chi2(3)      =     121,07
Log likelihood = -538,07992                     Prob > chi2       =     0,0000
------------------------------------------------------------------------------------
    Deleted deviating..| Coefficient  Std. err.      z    P>|z|     [95% conf. interval]
-----------------------+------------------------------------------------------------
 Self-rep Knowledge    |  -,4238069   ,1512332    -2,80   0,005    -,7202184   -,1273953
 Uncertainty (data)    |   ,4175588   ,0761608     5,48   0,000     ,2682863    ,5668312
 Peer Perception       |   ,9820384   ,1053028     9,33   0,000     ,7756486    1,188428
                 _cons |  -,2651773   ,1321579    -2,01   0,045    -,524202    -,0061525
-----------------------+------------------------------------------------------------
HighSchools            |
            var(_cons)|   ,1926978   ,1006623                      ,0692185    ,5364524
------------------------------------------------------------------------------------
LR test vs. logistic model: chibar2(01) = 10,85       Prob >= chibar2 = 0,0005
```

Regression results[1] from outcome variable

Copied shorter passages from other sources into your own text without marking them as quotes (0=no; 1=yes)

```
Mixed-effects logistic regression               Number of obs     =      1.218
Group variable: HighSchools                     Number of groups  =         51

                                                Obs per group:
                                                              min =          2
                                                              avg =       23,9
                                                              max =        138

Integration method: mvaghermite                 Integration pts.  =          7

                                                Wald chi2(8)      =     156,51
Log likelihood = -718,48024                     Prob > chi2       =     0,0000
------------------------------------------------------------------------------------
        Copied shorter | Coefficient  Std. err.      z    P>|z|     [95% conf. interval]
-----------------------+------------------------------------------------------------
Uncertainty (cit/plag) |   ,1771721   ,0689166     2,57   0,010     ,0420981     ,312246
Peer Perception        |   ,8065037   ,0849176     9,50   0,000     ,6400683    ,9729391
                       |
Courses not dedicated  |
to integrity issues    |  -,3547631   ,1577509    -2,25   0,025    -,6639492    -,045577
                       |
Country (ref. Denmark) |
              Ireland  |    1,47928   ,2300848     6,43   0,000     1,028322    1,930238
            Lithuania  |   1,137876   ,2434611     4,67   0,000     ,6607012    1,615051
             Portugal  |   1,097545   ,2278889     4,82   0,000     ,6508912    1,544199
         Switz_French  |  -,0911659   ,1796589    -0,51   0,612    -,4432909     ,260959
             Slovenia  |   ,6722432   ,2082317     3,23   0,001     ,2641166     1,08037
                       |
                 _cons |  -,1189564     ,14175    -0,84   0,401    -,3967814    ,1588685
-----------------------+------------------------------------------------------------
HighSchools            |
            var(_cons)|   1,29e-36   1,58e-19                             .           .
------------------------------------------------------------------------------------
LR test vs. logistic model: chi2(0) = 2,3e-13            Prob > chi2 =      .
```

[1] Final model shown after backward removal of statistically insignificant variables (p>0.05)

Regression results[1] from outcome variable

Added students as co-authors of group assignments even though they did not contribute (0=no; 1=yes)

```
Mixed-effects logistic regression               Number of obs     =      1.139
Group variable: HighSchools                     Number of groups  =         51

                                                Obs per group:
                                                              min =          2
                                                              avg =       22,3
                                                              max =        109

Integration method: mvaghermite                 Integration pts.  =          7

                                                Wald chi2(8)      =      82,11
Log likelihood = -718,57923                     Prob > chi2       =     0,0000
```

Added students	Coefficient	Std. err.	z	P>|z|	[95% conf.	interval]
Peer Perception	,3914742	,0813442	4,81	0,000	,2320426	,5509058
Gender(ref: male)						
Female	,4260349	,1374064	3,10	0,002	,1567232	,6953466
None of above/no answer	,4017474	,2530971	1,59	0,112	-,0943137	,8978085
Country (ref. Denmark)						
Ireland	-,7772229	,206034	-3,77	0,000	-1,181042	-,3734037
Lithuania	-,2201253	,2444895	-0,90	0,368	-,6993159	,2590652
Portugal	-,4161216	,2257395	-1,84	0,065	-,8585629	,0263197
Switz_French	-1,201492	,1900488	-6,32	0,000	-1,573981	-,8290032
Slovenia	-1,145692	,2217693	-5,17	0,000	-1,580352	-,7110323
_cons	,8121134	,1665653	4,88	0,000	,4856514	1,138575
HighSchools						
var(_cons)	1,50e-33	1,35e-17			.	.

```
LR test vs. logistic model: chi2(0) = 0,00              Prob > chi2 =      .
```

Regression results[1] from outcome variable

Received help from other students or family members on assignments you were supposed to complete on your own (0=no; 1=yes)

```
Mixed-effects logistic regression               Number of obs     =      1.168
Group variable: HighSchools                     Number of groups  =         51

                                                Obs per group:
                                                              min =          2
                                                              avg =       22,9
                                                              max =        121

Integration method: mvaghermite                 Integration pts.  =          7

                                                Wald chi2(3)      =      22,20
Log likelihood = -541,9215                      Prob > chi2       =     0,0001
```

Received help	Coefficient	Std. err.	z	P>|z|	[95% conf.	interval]
Uncertainty (coll/aut)	,1690328	,0829451	2,04	0,042	,0064635	,3316022
Peer Perception	,3382	,0986565	3,43	0,001	,1448369	,5315631
Discussions w. teachers						
outside regular classes	,5183049	,2282578	2,27	0,023	,0709279	,9656819
_cons	1,30814	,1123393	11,64	0,000	1,087959	1,528321
HighSchools						
var(_cons)	,056511	,0590071			,0073002	,4374544

```
LR test vs. logistic model: chibar2(01) = 1,63        Prob >= chibar2 = 0,1012
```
